# Sertraline, chlorprothixene, and chlorpromazine characteristically interact with the REST-binding site of the corepressor mSin3, showing medulloblastoma cell growth inhibitory activities

**DOI:** 10.1038/s41598-018-31852-1

**Published:** 2018-09-13

**Authors:** Jun-ichi Kurita, Yuuka Hirao, Hirofumi Nakano, Yoshifumi Fukunishi, Yoshifumi Nishimura

**Affiliations:** 1 Graduate School of Medical Life Science, Yokohama City University, 1-7-29 Suehiro-cho, Tsurumi-ku, Yokohama, 230-0045 Japan; 2 Laboratory for Chemistry and Life Science, Institute of Innovative Research, Tokyo Institute of Technology, 4259 Nagatsuda-cho, Midori-ku, Yokohama, 226-8503 Japan; 3 Molecular Profiling Research Center for Drug Discovery (molprof), National Institute of Advanced Industrial Science and Technology (AIST), 2-3-26, Aomi, Koto-ku, Tokyo, 135-0064 Japan; 4 Technology Research Association for Next-Generation Natural Products Chemistry, 2-3-26, Aomi, Koto-ku, Tokyo, 135-0064 Japan

## Abstract

Dysregulation of repressor-element 1 silencing transcription factor REST/NRSF is related to several neuropathies, including medulloblastoma, glioblastoma, Huntington’s disease, and neuropathic pain. Inhibitors of the interaction between the N-terminal repressor domain of REST/NRSF and the PAH1 domain of its corepressor mSin3 may ameliorate such neuropathies. *In-silico* screening based on the complex structure of REST/NRSF and mSin3 PAH1 yielded 52 active compounds, including approved neuropathic drugs. We investigated their binding affinity to PAH1 by NMR, and their inhibitory activity toward medulloblastoma cell growth. Interestingly, three antidepressant and antipsychotic medicines, sertraline, chlorprothixene, and chlorpromazine, were found to strongly bind to PAH1. Multivariate analysis based on NMR chemical shift changes in PAH1 residues induced by ligand binding was used to identify compound characteristics associated with cell growth inhibition. Active compounds showed a new chemo-type for inhibitors of the REST/NRSF-mSin3 interaction, raising the possibility of new therapies for neuropathies caused by dysregulation of REST/NRSF.

## Introduction

Repressor-element 1 silencing transcription factor (REST) or neural restrictive silencer factor (NRSF)^1,2^ was originally identified as a fundamental repressor, which binds to repressor-element 1 (*re1*) or neural restrictive silencer element (*nrse*) of many neuronal genes^3,4^ in both non-neuronal cells and neuronal progenitor cells. Subsequently, it was found to be expressed in pancreatic beta cells^5^ and various cancers as a tumor suppressor or oncogenic factor depending on the cell types^6,7^. Overexpression of REST/NRSF and/or dysregulation of its cellular expression pattern is related to several neuropathies, including medulloblastoma^8,9^ (a malignant pediatric brain tumor that originates in the cerebellum or posterior fossa^10^), glioblastoma^11,12^, Huntington’s disease^13–16^, neuropathic pain^17,18^, and Parkinson’s disease^19^, as well as potentially autism^20^ and fibromyalgia^21^.

REST/NRSF mediates transcriptional repression by the recruitment of two corepressor complexes: mSin3 at its N-terminus, and CoREST plus the histone H3K9 methylase G9a at its C-terminus^22^. The mSin3 complex contains two histone deacetylases, HDAC1 and HDAC2^23^, and has been recently implicated in cancer as an important epigenetic regulator^24^. mSin3 contains four paired amphipathic helix domains (PAH1–PAH4), of which PAH1 is responsible for interacting with the N-terminal repressor domain of REST/NRSF^25^. Although the repressor domain is intrinsically disordered in the unbound state, NMR has shown that it forms a helix consisting of about ten amino acids after binding to the PAH1 domain of mSin3B^25^, an isoform of mSin3. In the NMR structure of the complex, the short helix of NRSF/REST is deeply buried in the hydrophobic groove of PAH1. Compounds that bind to the mSin3B PAH1 groove are presumably inhibitors of REST/NRSF function^21,25^, and thus potentially drug candidates to ameliorate disorders originating from up-regulation of REST/NRSF^21,26–29^.

Here, to develop new inhibitors with different chemo-types from those so far examined, we first performed *in-silico* drug screening of nearly 2 million commercially available compounds and approved neuropathic drugs that are expected to overcome blood–brain–barrier (BBB) limits, yielding 52 compounds that potentially bind to the mSin3 PAH1 domain. The binding ability of the 52 compounds was examined by NMR screening methods^30^, including two ligand-based screening methods, saturation transfer difference (STD)^31,32^ and WaterLOGSY^33,34^, and one protein-based screening method, heteronuclear single quantum coherence (HSQC), while their inhibitor activity was examined by using a medulloblastoma cell line, DAOY^35–37^.

Next, we tried to identify a correlation between the characteristic binding mode of a compound to REST/NRSF and its DAOY cell growth inhibitory activity, using both principal component analysis (PCA)^38–40^, and sparse partial least square discriminant analysis (sPLS-DA)^41^. Lastly, we obtained the NMR-docking structures of two of the identified active compounds (sertraline and chlorpromazine), on the mSin3B PAH1 domain based on their chemical shift perturbations (CSPs) and compared them with the binding mode of sertraline to a serotonin transporter.

## Results

### *In-silico* screening for inhibitors of the mSin3–REST/NRSF interaction

To identify potential inhibitors of the interaction between mSin3 and REST/NRSF, we performed two types of *in-silico* screening: ligand-based drug screening (LBDS) to identify compounds similar to known active compounds; and structure-based drug screening (SBDS) based on the target protein structure to identify new active chemo-types (scaffolds) that differ from the chemo-types of known active compounds. We applied our software myPresto (freely available from https://www.mypresto5.jp/en/) to screen compounds from the KEGG DRUG database (http://www.kegg.jp/kegg/drug/)^42^ of approved drugs, and approximately 2-million commercially available compounds selected from the LigandBox database. For the SBDS, a molecular dynamics simulation generated protein structures in water based on an initial structure obtained from the PDB (PDB ID:2CZY).

Among the approved drugs, we focused on drugs for the central nerve system (CNS) because these drugs penetrate the BBB, which can be a major obstacle in drug therapy. For the same reason, we restricted the molecular weight of compounds from the LigandBox database to less than 350 Da because, in general, the transport of smaller compounds across the BBB is faster than that of larger compounds. Ultimately, the screening process yielded 52 compounds that were potential inhibitors of the REST/NRSF interaction with mSin3 (Supplementary Fig. S1) and the 52 compounds were commercially obtained (Supplementary Table S1). In Table S1, compounds 1–23 and compounds 24–52 were from the LigandBox database and KEGG DRUG database, respectively.

### Evaluation of PAH1 binding affinity by NMR titration

The ability of the 52 compounds to bind to the mSin3B PAH1 domain were examined by using STD and WaterLOGSY NMR experiments. Because the mSin3B PAH1 domain has a small molecular weight that would not be expected to sufficiently transfer spin diffusion to the ligand, both experiments were performed with a GST fusion protein of PAH1. First, the binding activity was approximately evaluated by the ligand signal intensity ratio of each experiment to the bulk ligand intensity. Next, we performed an HSQC titration experiment to obtain more detailed information of the interaction at residue-specific resolution (Supplementary Fig. S2b) with reference to the HSQC spectrum of unbound PAH1 with amino acid assignments (Supplementary Fig. S2a).

**Figure 1:**
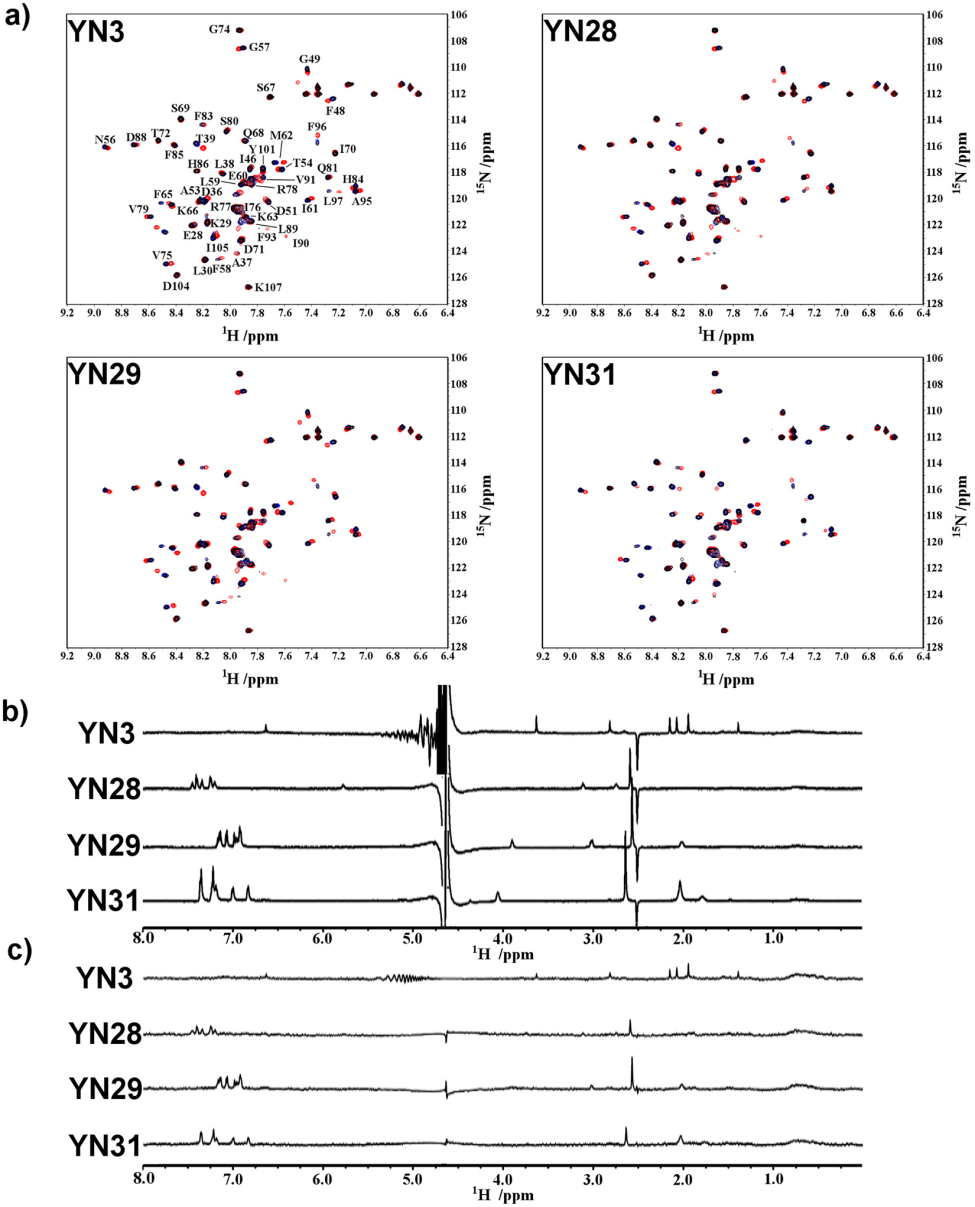
NMR spectra of YN3, YN28, YN29, and YN31. (**a**) HSQC ligand titration experiment of mSin3B PAH1 with each compound. In each spectrum, black signals correspond to 100 μM free PAH1 domain, and blue and red signals correspond to the additions of 100 μM and 1 mM ligand, respectively. In the YN3 spectrum, the assigned amide signals of PAH1 are shown. (**b**) WaterLOGSY spectrum of each compound. (**c**) STD spectrum of each compound.

The HSQC spectra indicated that four compounds YN29, YN31, YN3, and YN28, have a strong affinity for the mSin3B PAH1 domain (Fig. 1). All four compounds showed significant signals in both WaterLOGSY and STD spectra (Fig. 1). It was difficult to estimate the Kd values for these compounds directly from HSQC titration experiments because of their relatively strong binding affinities. Thus, the Kd value for the specific binding of each ligand was obtained by WaterLOGSY titration experiments at a protein concentration of 1 μM and a ligand concentration ranging from 5 to 50 μM. Although these solution conditions are very dilute in terms of NMR, Kd values were obtained for YN28 (35 ± 11 μM), YN29 (15 ± 4.2 μM), and YN31 (39 ± 22 μM) (Supplementary Fig. S2(e)). However, the sensitivity of the WaterLOGSY spectrum of YN3 under such dilute conditions was too poor to obtain a Kd value. Weak but significant HSQC spectral changes were observed for seven compounds, YN25, YN26, YN37, YN38, YN39, YN40, and YN42. All seven compounds showed relatively strong signals in the WaterLOGSY spectra; however, only YN39, YN40, and YN42 showed clear signals in the STD spectra, and YN25, YN26, YN37, and YN38 did not (Supplementary Fig. S2). The mSin3B PAH1 domain has a shallow ligand-binding pocket, which water molecules can access. Thus, bound water molecules located around each bound ligand will contribute directly to enhancement of WaterLOGSY signals; in STD experiments, however, the bound water molecules will reduce magnetization saturation of the protein moiety because saturated bound water molecules exchange rapidly with bulk waters. For this reason, the relatively weak binding ligands, YN25, YN26, YN37, and YN38 showed significant WaterLOGSY signals but poor STD signals in H_2_O solution.

### Structure–activity relationship of the active compounds

**Figure 2:**
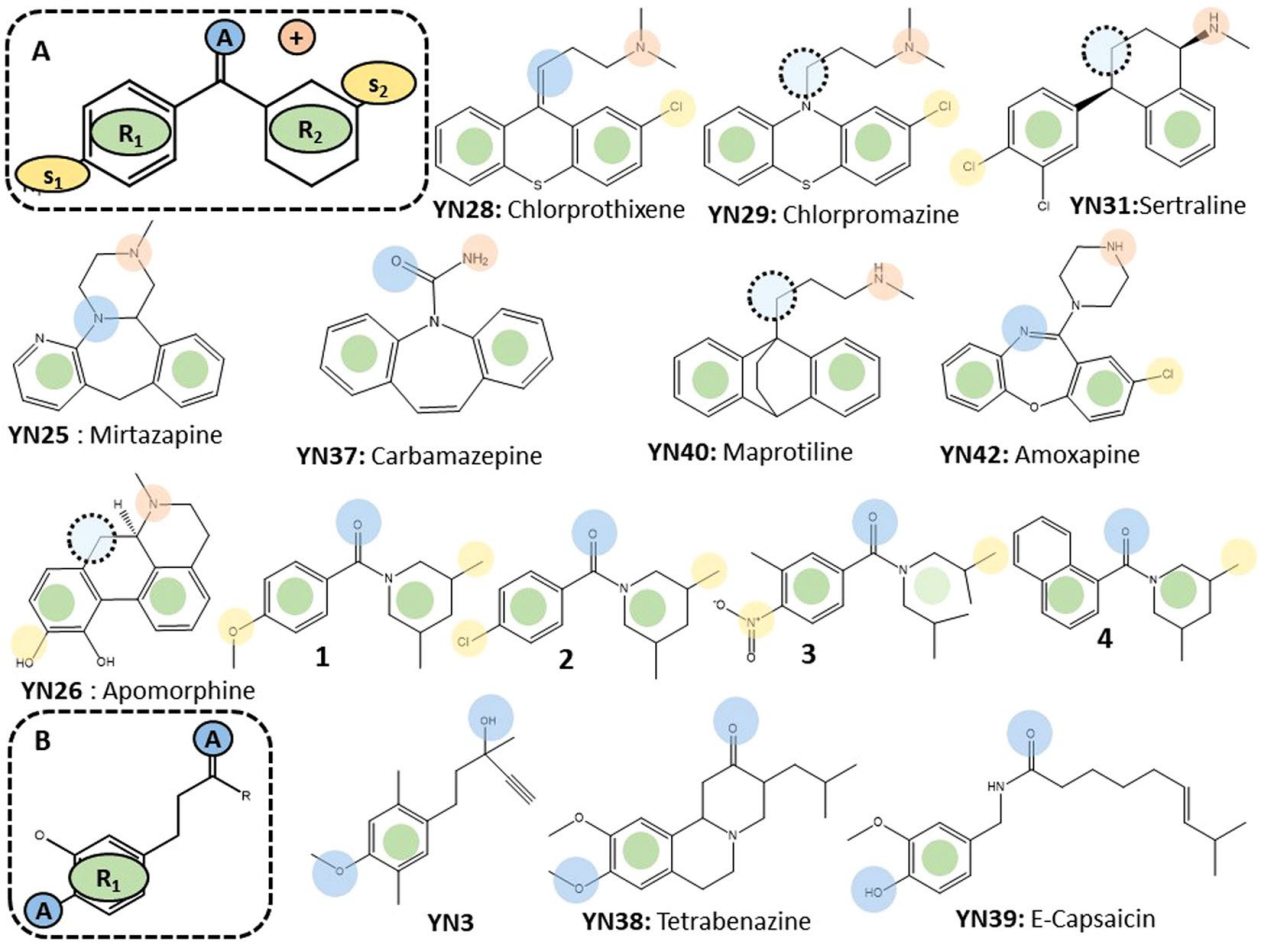
Structure–activity relationship of 11 potential mSin3 inhibitors. The top dashed box (**A**) represents pharmacophore **A**. R1 (green), R2 (green), A (blue), s1 (yellow), s2 (yellow), and + (orange) indicate two rings, hydrogen acceptor, two side chains, and positive charge, respectively. Dotted circles represent mismatch sites. The bottom dashed box (**B**) represents pharmacophore **B**.

The NMR experiments suggested that compounds YN29, YN31, YN3, and YN28 showed strong binding affinity, and compounds YN25, YN26, YN37, YN38, YN39, YN40, and YN42 showed moderate binding affinity to the mSin3B PAH1 domain. Figure 2 shows the structure–activity relationship (SAR) of these 11 compounds. Two pharmacophores were found to represent the different SARs: namely, pharmacophore **A** (panel A in Fig. 2), and pharmacophore **B** (panel B in Fig. 2). Pharmacophore **A** was a major SAR that was present in 12 compounds (YN25, YN26, YN28, YN29, YN31, YN37, YN40, YN42, and compounds **1–4**, which are active compounds previously reported by us using the same assay system as the present study^29^), whereas pharmacophore **B** was contained in only three compounds (YN3, YN38, and YN39). Thus, below we discuss only pharmacophore **A**.

All binding compounds consist of two rings except for compound **3**, in which two chains extending from the amide may be folded to occupy a volume similar to that occupied by one 6-membered ring. Thus, all inhibitor compounds belonging to pharmacophore **A** are likely to maintain a common structural feature: that is, a structure consisting of two 6-membered ring groups connected by a short linker, as found in previous compounds. In many cases, the linker has a hydrogen-bond acceptor atom, and the two small ring systems have one or two small side chains. Some of the ligands have cationic amine nitrogen atoms, and some ligands have one nitrogen atom.

Except for this simple structural feature, there was no significant structural similarity between the inhibitors. The set of compounds showed a narrow SAR. We note that most approved drugs for CNS have amine nitrogen atoms because they target the monoamine receptor and utilize cation transporters to penetrate the BBB. Thus, the CNS compound database shows bias toward amine nitrogen-containing compounds, we cannot draw conclusions on the importance of the amine nitrogen atom in the protein–ligand interaction.

### 3D growth inhibition assays of medulloblastoma

**Figure 3:**
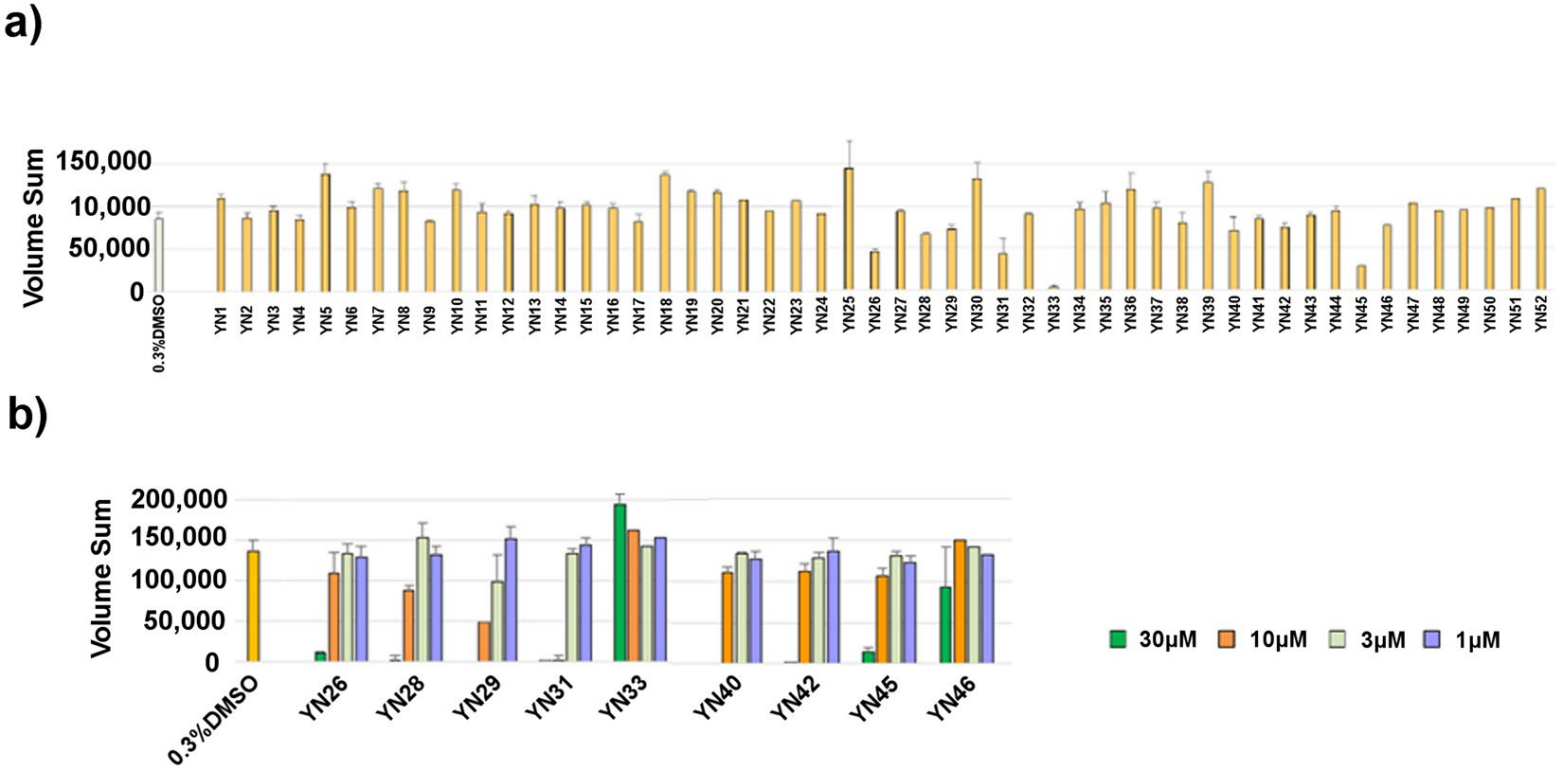
Effect of YN compounds on 3D spheroid growth of DAOY medulloblastoma cells. DAOY cells were seeded at a density of 1000 cells in 100 μl of medium into ultra-low attachment 96-well U bottom plates. Next, 10 µL of each YN compounds (300 μM in 3% DMSO), or medium was added to triplicate wells on day 1. Spheroid growth was monitored over time in a non-destructive way by a Cell3iMager scanner and scored as volume sum (volume and optical density of the single sphere). (**a**) Primary screening data illustrating the effect of 52 YN compounds at a concentration of 30 μM on 3D cell growth. (**b**) Secondary screening of active compounds at a concentration of 30, 10, 3 and 1 μM. Data after 6 days of incubation are shown.

NRSF/REST is expressed in various cancers and plays a role in medulloblastoma tumorigenesis by blocking the differentiation of stem-like cells^8^. Relative to 2D culture, tumor cells grown in three-dimensional (3D) culture have greater analogy to naturally occurring cells and show a phenotype of cancer stem cells^43^. Thus, we constructed a new forced-floating 3D culture method to generate spheroids of the DAOY human medulloblastoma cell line (ATCC)^44^. The 52 compounds were tested at a concentration of 10 μM against DAOY spheroids formed in 96-well culture plates (Fig. 3a). Compounds showing activity were tested at additional concentrations in a second assay (Fig. 3b).

Compounds YN28, YN29 and YN31, which had strong binding activity in the NMR experiments, showed strong DAOY growth inhibitory activity with IC_50_ values of 9.1 μM, 4.5 μM, and 5.1 μM, respectively. However, compound YN3, which has also had strong binding activity, did not show inhibitory activity on DAOY growth. In addition, compounds YN26, YN40, YN42 and YN45, which showed modest binding activity by NMR, showed relatively strong DAOY growth inhibitory activity with IC_50_ values of 18 μM, 15 μM, 16 μM, and 14 μM, respectively.

These findings suggest that the growth inhibitory activity of each compound is somewhat related to the binding activity observed in NMR experiments; however, the relation is not strict. For example, YN3 had a strong binding activity, but showed no DAOY inhibitory activity. Next, therefore, we examined in detail the PAH1 CSPs in each HSQC spectrum induced by ligand binding in a residue-specific manner in order to derive a plausible correlation between amino acid residues of PAH1 perturbed by a ligand binding and medulloblastoma cell growth inhibitory activity.

### Correlation of CSPs and cell growth inhibitory activity by multivariate analysis

Multivariate statistical analysis using amino acid CSPs as variables was applied to the HSQC CSPs of PAH1 induced by each ligand binding in order to clarify effective and ineffective ligands with respect to cell activity. For this purpose, we defined IC_50_ < 30 μM for effective and IC_50_ > 30 μM for ineffective ligands on the basis of the DAOY cells growth inhibitory assay.

#### Unsupervised analysis

Initially, we applied principal component analysis (PCA) to the CSPs of 55 amino acids of PAH1 as an unsupervised pre-processing step. In PCA, the first five components explained 58% of variance of the 55 variables. However, the defined effective and ineffective groups seemed to overlap considerably in the two-dimensional score plots of any pair of the first five components (Supplementary Figs S3 and S4a). In addition, it was difficult to differentiate the strong binding compounds with effective DAOY growth inhibitory activity (YN28, YN29 and YN31) from the strong binding compound with ineffective DAOY growth inhibitory activity (YN3) by PCA. It is noteworthy that YN28, YN29 and YN31 are approved CNS drugs but YN3 is not, and we have not tested whether YN3 can enter DAOY cell or not. Furthermore, it was difficult to analyze the dispersion factors in PCA, because almost all variables contributed to each dimension with small values.

As an alternative to PCA, the sparse vectors method can be applied to multivariate analysis^41,45–47^ to improve the separation between groups by eliminating non-essential variables that contribute to intra-group dispersion via lasso penalizations or similar. In sparse PCA (sPCA)^45,46^ (Supplementary Fig. S4b) with sparse loading vectors (Supplementary Fig. S4c), however, the effective and ineffective groups were not separated clearly, although structural information could be obtained from sparse loading vectors.

Sparse loading plots, in which each component consists of contributions from variables corresponding to characteristic amino acid residues, were used to identify amino acids with contributions of more than 0.3 to each component. These amino acids were then mapped on the REST/NRSF contact surface of PAH1 (PDB code: 2CZY, Supplementary Fig. S4d). The main contribution to component 1 came from Met62, Leu59, Thr39, Lys66, and Arg77. These residues are on the REST/NRSF contact surface of mSin3B PAH1, suggesting that component 1 is characterized as strength of ligand binding to the pocket. However, the other components of sPCA provided no clear structural information.

#### Supervised analysis

**Figure 4:**
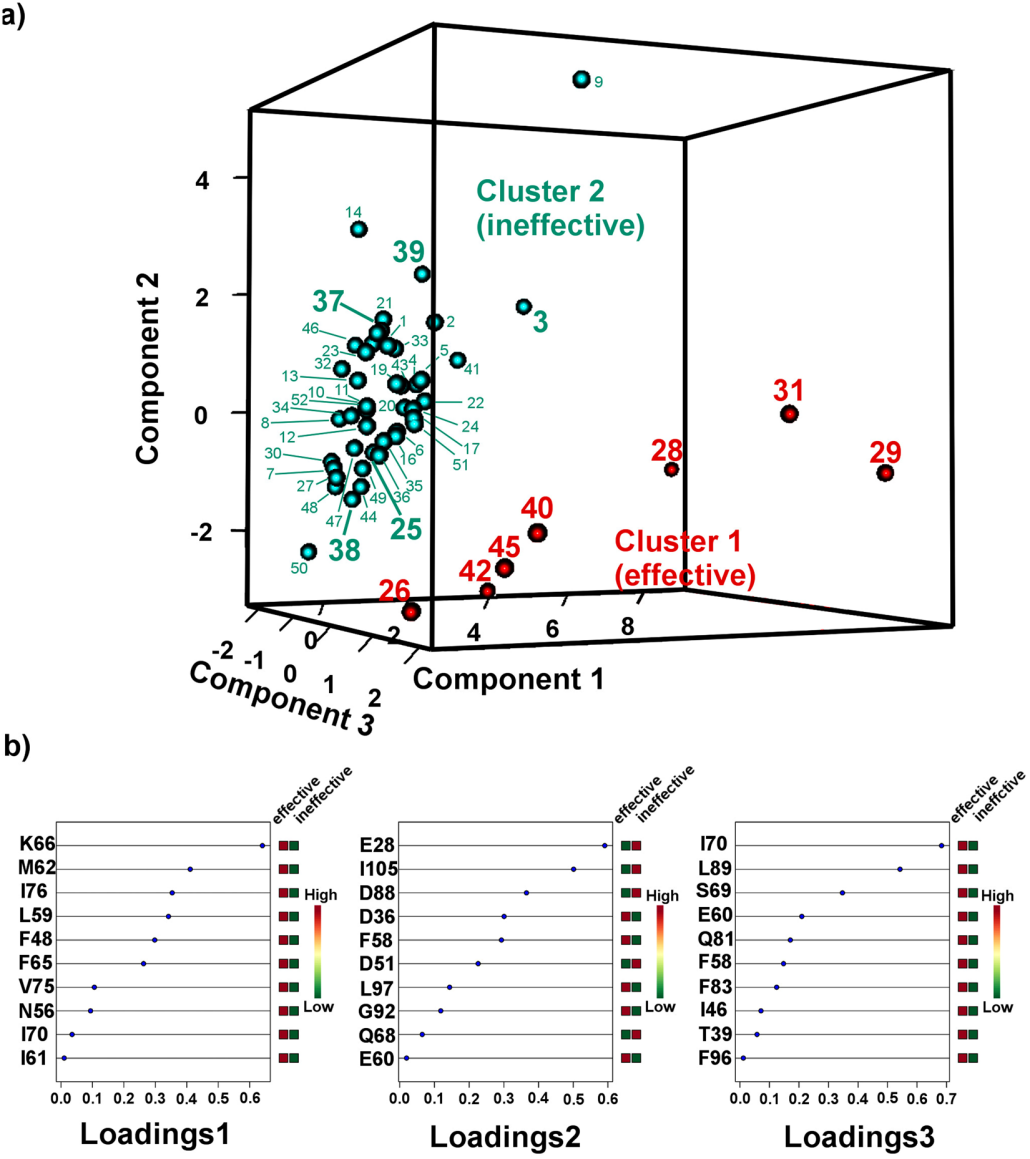
Application of sPLS-DA to CSPs. (**a**) 3D score plot of sPLS-DA. Red solid circles and green solid circles indicate, respectively, the effective and ineffective compounds for DAOY cell growth inhibition. (**b**) Sparse loadings of first three components.

To overcome the considerable overlap between groups in the PCA and sPCA models, we performed sparse partial least square discriminant analysis (sPLS-DA)^41^ to differentiate between the effective and ineffective ligands in a supervised way. In this analysis, the two clusters were relatively well separated in the PC1/PC2 two-dimensional score plot (Supplementary Fig. S5), and more clearly separated in the PC1/PC2/PC3 three-dimensional score plot (Fig. 4a) with sparse loading (Fig. 4b). This result was supported by the SAR; in other words, the compounds corresponding to pharmacophores **A** and **B** were contained in the major and minor clusters, respectively. Thus, the two clusters should correspond to the different pharmacophores of mSin3.

In addition, to evaluate this method, we performed a 7-fold cross validation with three different distance prediction methods: maximum distance, centroid distance, and Mahalanobis distance. In the maximum distance method, the estimate of the sPLS-DA classification error rate converged to about 8% in three dimensions (Supplementary Fig. S6), suggesting that sPLS-DA has sufficient prediction in three dimensions.

#### Sparse loading vectors of sPLS-DA and structural mapping

**Figure 5:**
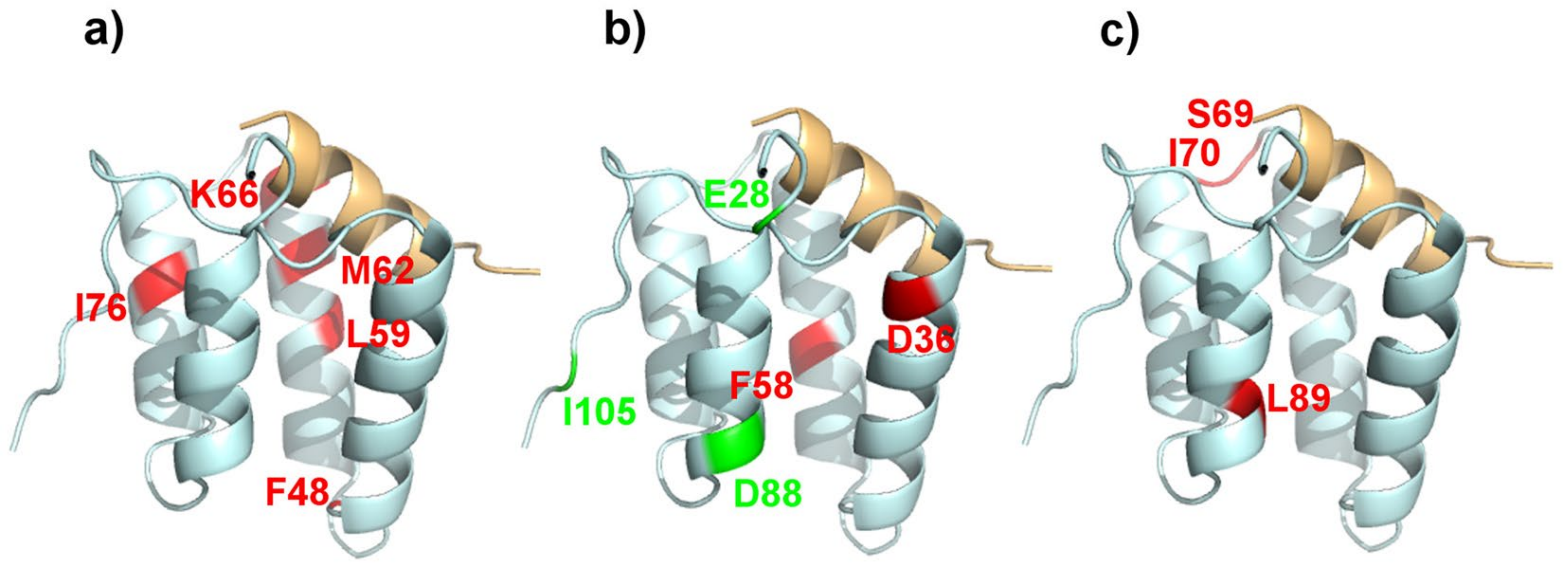
Sparse loadings of the first three components of sPLS-DA mapped on the PAH1–REST/NRSF complex (PDB code: 2CZY). (**a**–**c**) correspond to sparse loadings of components 1, 2 and 3, respectively. Sparse loadings with a positive correlation for DAOY growth inhibitory activity are shown in red; those with negative correlations are shown in green.

Next, we mapped amino acids with contributions of more than 0.3 to each component in the sparse loading plots on the mSin3B PAH1–REST/NRSF complex (PDB code: 2CZY) (Fig. 5). The main contributions to component 1 came from Lys66, Met62, Ile76, Leu59, Phe48 and Phe65. These residues are on the REST/NRSF-contact surface of PAH1, suggesting that, similar to sPCA, component 1 is characterized as strength of ligand binding to the pocket. In addition, these loadings were positively correlated with compound effectiveness in the cell growth inhibition assay.

By contrast, the main contribution to component 2 came from Glu28, Ile105, Asp88, Asp36, and Phe58, residues that are located close to the tail or linker. The first three amino acids (Glu28, Ile105, and Asp88), which are positioned far from the REST/NRSF contact surface of PAH1, were negatively correlated with compound effectiveness in the cell growth inhibition assay, suggesting that component 2 is characterized as non-specific bindings to the mSin3B PAH1. In contrast, the last two residues (Asp36 and Phe58), which are located on the contact surface with the neighboring helix, were positively correlated with compound effectiveness in the cell growth inhibition assay suggesting that these two residues are involved in the rearrangement of helix I of PAH1. Thus, component 2 is characterized as a mixed indicator of helix rearrangement and non-specific binding to the mSin3B PAH1 domain.

The main contribution of component 3 came from Ile70, Leu89, and Ser69, which are located in the linker relatively close to the REST/NRSF-contact surface of PAH1, suggesting that this component is characterized as the structural changes of PAH1 after ligand binding. In addition, these residues were positively correlated with compound effectiveness in the cell growth inhibition assay, as in component 1.

In fact, two NMR structures (PDB code: 2CR7 and 2CZY) have revealed that the PAH1 domain of mSin3B shows a large structural change when it binds to REST/NRSF. In a previous prediction of CSPs due to this structural change using SHIFTX2^48^, we found that the perturbations around Ile70 were the largest^21^, which suggests that the active compounds identified here cause structural changes in PAH1 similar to those caused by REST/NRSF.

### NMR data-guided docking calculation

**Figure 6:**
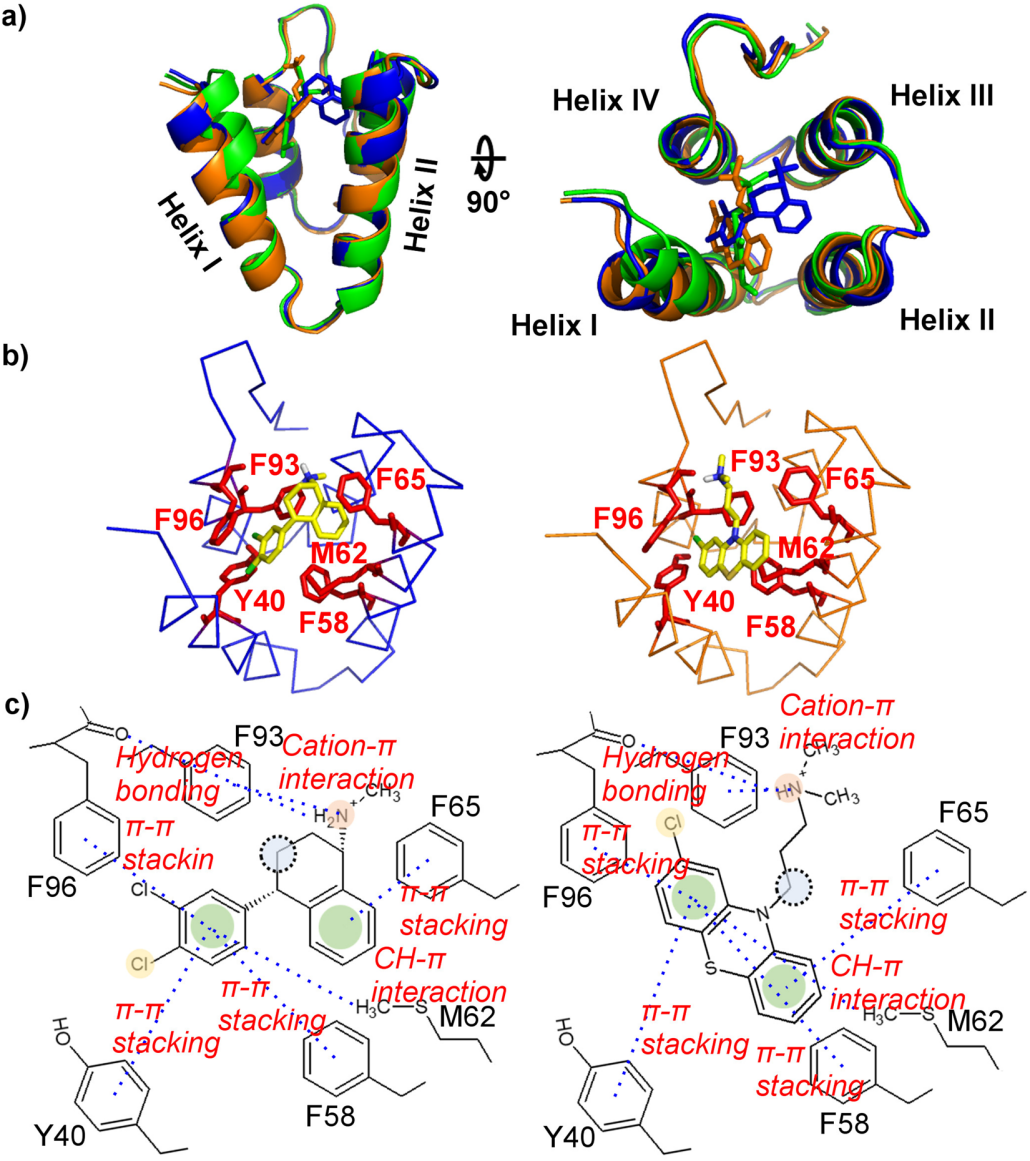
Structural comparison of PAH1–YN3, PAH1–chlorpromazine, and PAH1–sertraline interactions. (**a**) Superimpose docking structures of PAH1–YN3 (green), PAH1–chlorpromazine (orange), and PAH1–sertraline (blue) complexes in cartoon representation. For these docking calculations, the PAH1 structure of 2CZY was used as the initial structure. (**b**) Structural comparison of PAH1–chlorpromazine (right, orange) and PAH1–sertraline (left, blue). (**c**) Chemical schematic drawing of interactions in the PAH1–chlorpromazine and PAH1–sertraline complexes.

We used NMR data-guided docking to clarify the differences in complex structure between effective and ineffective compounds that showed strong binding to mSin3B. YN29 (chlorpromazine, an antipsychotic drug) and YN31 (sertraline, an antidepressant and serotonin reuptake inhibitor) were selected as typical effective compounds, while YN3 was selected as a binding but ineffective compound. Each docking structure was calculated by using the HADDOCK/CNS docking protocol^49–51^ (Fig. 6).

The most significant difference between the DAOY cell-effective (chlorpromazine and sertraline) and cell-ineffective (YN3) complexes is the arrangement of helix I. In the case of the two effective compounds, helix I is oriented outwards, which opens the pocket, whereas in the case of the ineffective compound, helix I is oriented more inwards, closing the pocket. This suggests that the two effective compounds (chlorpromazine and sertraline) are bulkier than YN3 and cannot bind without rearrangement of helix I, consistent with the results of multivariate analysis. In addition, the docking pose of pharmacophore **A** was consisted with the SAR: Phe58, Tyr40, Phe65 and Phe96 of PAH1 show π-π stacking with the two aromatic rings of the ligands. The carboxyl group of the main chain of Phe96 forms a hydrogen bond with the amine of the ligand. There are small cavities for s1 and s2 of pharmacophore **A**. Although chlorpromazine with its bulky three rings is buried in the pocket slightly deeper as compared with sertraline, both ligands bind to the pocket in a similar manner, including the spatial arrangement of the positive charges of the amine group and the aromatic rings. These complex structures suggest that both the mode of binding to the pocket and the rearrangement of helix I are important factors in the inhibition of DAOY cell growth.

## Discussion and Conclusion

Our in-slico screening coupled with NMR titration identified several compounds that interact with the PAH domain of mSin3B. SAR analysis of these active compounds as well as SAR analysis of known active compounds were consistent with the present findings (Fig. 2). In other words, the active compounds contained at least two rings linked by one or two atoms, and the rings may have short branches and in most cases the linker contains an amino-type nitrogen atom, which might act as a hydrogen bond acceptor. The SAR analysis was further supported by our mSin3B–compound docking study, in which common features of the active compounds were found to interact with mSin3B. Thus, these compounds are likely to be active, although their chemo-type (scaffold) is completely different from that of a previously reported active compound consisting of an α-helix peptide mimic middle molecule^21^.

Although we focused on pharmacophore **A** in the present study, we also observed a second pharmacophore, pharmacophore **B** (Fig. 2). The two SARs correspond to the clusters obtained by the sPLA-DA analysis. In other words, pharmacophores **A** and **B** should prompt different changes in protein structure corresponding to clusters 1 and 2, respectively, in the sPLA-DA analysis. Our docking conformation analysis in the protein-ligand docking study further supported this assumption. In addition, the two clusters showed different biological phenotypes: compounds in cluster 1 showed growth inhibition of DAOY cells, whereas those in cluster 2 did not inhibit cell growth. Collectively, these results suggest that the NMR-based sPLA-DA analysis might be useful as a functional assay.

Interestingly, antidepressant or antipsychotic medicines, including sertraline, chlorprothixene, and chlorpromazine were found to strongly bind to PAH1; sertraline is a selective serotonin reuptake inhibitor that is known to be highly selective for the serotonin transporter (SERT). Notably, the major target protein of the approved drugs identified in this study was neither mSin3 nor REST/NRSF. Rather, the common targets of the active ligands were the sodium-dependent serotonin transporter and dopamine receptors, as reported and summarized in DrugBank (https://www.drugbank.ca/)^52^. This observation suggests that these proteins may share similar ligand-binding conformations. To our knowledge, however, there is no homology between mSin3 and the serotonin transporter or dopamine receptors.

**Figure 7:**
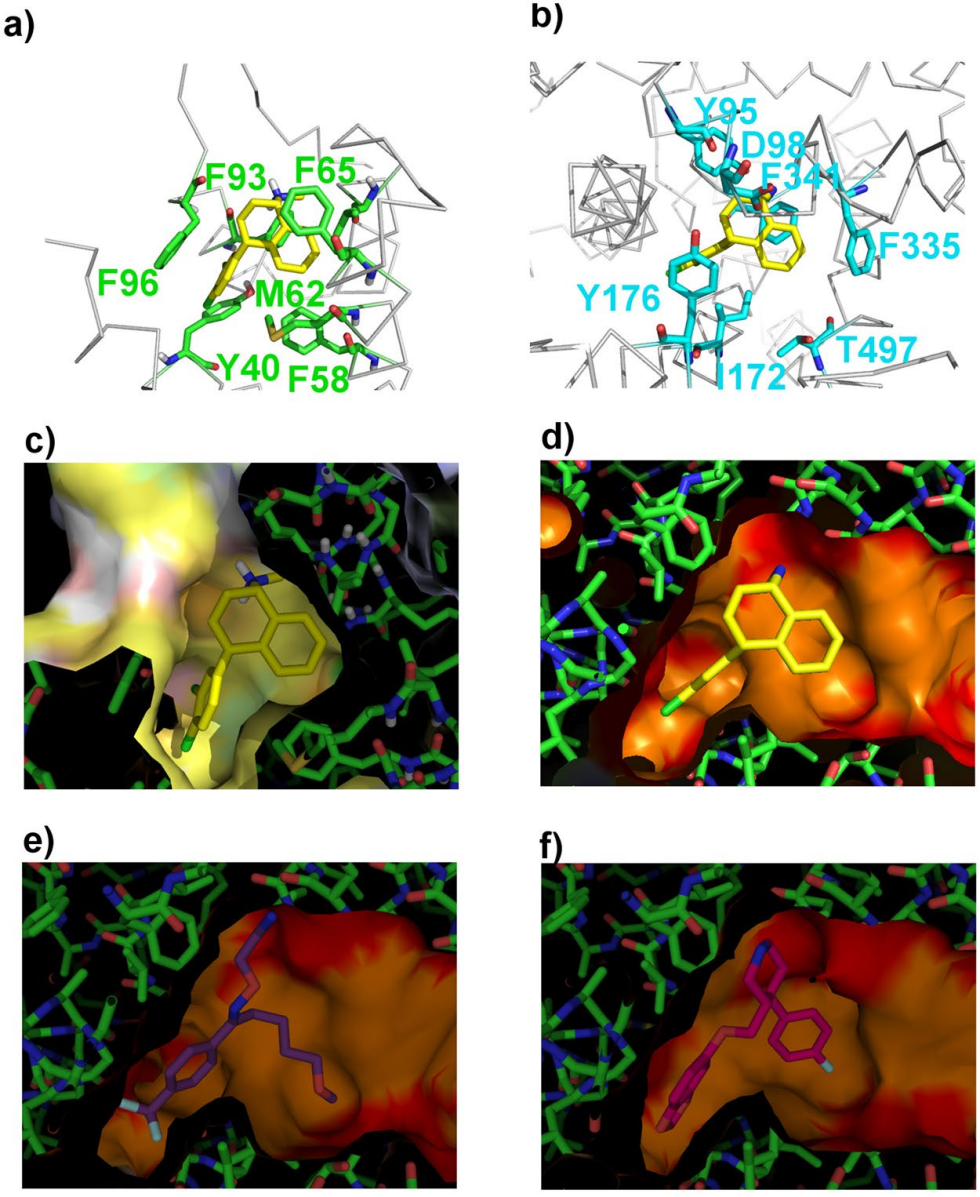
Structural comparison of the binding pockets of PAH1 and the serotonin transporter. (**a**) Docking structure of PAH1–sertraline represented as a ribbon model. Important residues in the interaction are represented as a stick model. Sertraline atoms are colored by type (yellow, carbon; white, hydrogen; blue, nitrogen; green, chlorine). (**b**) X-ray structure of the serotonin transporter (SERT)–sertraline complex (PDB code: 6AWO). Important residues in the interaction are represented as a stick model. Sertraline atoms are colored by type as in panel (a). (**c**) Binding site structure of the PAH1–sertraline docking calculation represented as a surface model using eF-surf ^78^, colored by default. (**d**–**f**) Binding site structures of 6AWO, 6AWP and 6AWN, respectively, represented as a surface model using eF-surf, colored by default.

Recently, mechanisms underlying the recognition of three antidepressants, sertraline, fluvoxamine, and paroxetine, by SERT have been reported on the basis of crystal structures^53^. We therefore compared the binding structures of sertraline to SERT and mSin3B as shown in Fig. 7.

Although no amino acid sequence homology between SERT and mSin3B PAH1 domain was identified, both binding pockets contained similar amino acids in similar positions. Sertraline interacts with Asp98, Tyr95 and Phe341 of SERT and with the Phe96 main chain and Phe93 of PAH1 via its amino group (+); with Tyr176 and Ile172 of SERT and with Tyr40, Phe58 and Phe96 of PAH1 via its R1 aromatic ring; with Phe335 of SERT and with Phe65 of PAH1 via its R2 ring. The antidepressant fluvoxamine binds strongly to SERT but not so strongly to PAH1. Fluvoxamine has no ring corresponding to R2, which might reduce its affinity for PAH1. Another antidepressant, paroxetine, binds strongly to SERT by a binding mode similar to that of sertraline; therefore, we examined the mSin3B binding activity of paroxetine. STD, WaterLOGSY, and HSQC experiments showed that paroxetine binds strongly to the PAH1 domain of mSin3B (Supplementary Fig. S7). Collectively, these findings reveal that some antidepressants that bind to SERT have affinity for the mSin3B PAH1 domain. Interestingly, several different types of epigenetic mechanisms, including key REST/NRSF effectors such as histone acetylation/methylation and miRNAs, have been reported for antidepressants^54^.

Our data suggest that the currently approved CNS drugs, sertraline, chlorprothixene, and chlorpromazine are likely to have polypharmacological activity against the mSin3B PAH1 domain, which binds to REST/NRSF, whose dysregulation is related to glioblastoma, Huntington’s disease, and neuropathic pain in addition to medulloblastoma. Several studies have suggested that sertraline or chlorpromazine is effective against glioblastoma, either when used alone or when used as combinatorial chemotherapy with other drugs^55–60^. Although previous studies have proposed other mechanisms of tumor suppression by sertraline or chlorpromazine, our present findings may provide new clues for clinical research on sertraline or chlorpromazine against glioblastoma. Sertraline has also been reported to have positive effects in Huntington’s disease models via several different mechanisms^61,62^.

As far as we know, our study is the first to demonstrate the activities of these drugs against medulloblastoma cells. Medulloblastoma is the most common malignant brain tumor in children. Current therapies for medulloblastoma impose debilitating side effects on the developing child. We found here that several approved neuropathic drugs induced NMR chemical shift change in the mSin3B PAH1 domain indicative of binding and also inhibited 3D spherical growth of the medulloblastoma cell line DAOY. In particular, sertraline and chlorpromazine showed strong binding to the PAH1 domain and high growth inhibition of DAOY cells. Collectively, our findings raise the possibility that drug repositioning might aid in the therapeutically unmet need for medulloblastoma, especially because approved neuropathic drugs have BBB permeability and human oral bioavailability.

## Methods

### *In-silico* screening

For the SBDS, MolSite was used to select the ligand-binding sites of the mSin3 structure^63^. Next, based on the protein structure, multiple-target screening (MTS) was used to dock compounds selected from a database to proteins, including the target and other proteins, to generate a protein–compound affinity matrix^64^. A machine-learning MTS (ML-MTS) method approximates the binding affinity of each compound to the target protein by a linear combination of many docking scores from the reference proteins^64^. For the LBDS, molecular-dynamics maximum-volume overlap (MD-MVO) was used to select structurally similar compounds from a given compound database^65^.

The protein–compound docking scores used in the MTS and ML-MTS drug screenings were calculated by the protein–compound docking program Sievgene^66^. The computational setup was the same as in our previous study^64^. In brief, Sievgene generated up to 100 conformers for each compound, and the potential grid was 60 × 60 × 60 for all proteins. Each edge length of the grid was about 35–45 Å. The docking-scoring function is based on the physical chemistry (accessible surface area, van der Waals potential, and electrostatic potential).

We performed a protein–compound docking simulation based on the soluble protein structures registered in the PDB. The probe protein set consisted of 188 arbitrarily selected protein structures, as in our previous study^64^. All of these structures were protein−ligand complexes. The structure of mSin3B (PDB code: 2CZY) was added to the protein set as the target protein^25^.

For the protein set used in the MTS and ML-MTS drug screening, complexes containing a covalent bond between the protein and ligand were removed, and all missing hydrogen atoms were added to form all-atom models of the proteins. All water molecules and cofactors were removed from the protein structures. All Asp and Glu residues were prepared as negatively charged forms, whereas Lys and Arg residues were prepared as positively charged forms. The atomic charges of the proteins were the same as those in AMBER parm99^67^. The docking pocket of each protein was indicated by the coordinates of the original ligand.

The 3D structures of the compounds were energy-optimized by cosgene/myPresto^68^ using the general AMBER force field (GAFF)^69^. The atomic charges were calculated by the MOPAC AM1 model using the Hgene program of the myPresto suite. Each functional group in all molecules was set to the dominant ionic form at pH 7.

### Sample preparation

#### mSin3B PAH1

The PAH1 domain of mouse Sin3B (mSin3B PAH1; a.a. 28–107 with a Met at the N-terminus) was obtained as described^23,25^. The coding region of PAH1 was subcloned into the pET28a vector (Novagen) to produce a His6-tagged protein with a recombinant thrombin protease recognition site. The protein was expressed in *Escherichia coli* strain BL21(DE3) grown at 37 °C in M9 minimal media containing ^15^NH_4_Cl (0.15%). When the absorbance at 600 nm reached 0.5–0.6, 1 mM isopropyl-1-thio-β-D-galactopyranoside (IPTG) was added to induce protein expression. After an additional 16 h growth at 20 °C, the cells were harvested and resuspended in 50 mM sodium phosphate buffer (pH 8.0, 300 mM NaCl and 10 mM imidazole). The cells were lysed by sonication on ice and centrifuged (39,000 g). The supernatant was loaded onto a nickel/nitrilotriacetic acid/agarose (Qiagen) column. The column was washed with 50 mM sodium phosphate buffer (pH 8.0, 300 mM NaCl and 10 mM imidazole), and the protein was eluted by a linear gradient from 10 to 250 mM imidazole. After a concentration step, the buffer was changed to 20 mM Tris-HCl buffer (pH 8.0, 2.5 mM CaCl_2_ and 150 mM NaCl). The protein was digested with thrombin for 16 h at room temperature to remove the His_6_ tag, and purified by gel-filtration column chromatography (Superdex30; Pharmacia) with 20 mM potassium phosphate buffer (pH 7.5, 150 mM NaCl).

#### GST fusion mSin3B PAH1

The coding region of mSin3B PAH1 was subcloned into the pGX-6P-1 vector (GE Healthcare) to produce a GST fusion protein with a recombinant PreScission protease recognition site by using Genscript’s gene synthesis service. The protein was expressed in *Escherichia coli* strain BL21(DE3) grown at 37 °C in LB medium. When the absorbance at 600 nm reached 0.5–0.6, 1 mM isopropyl-1-thio-β-D-galactopyranoside (IPTG) was added to induce the protein expression. After an additional 16 h growth at 15 °C, the cells were harvested and resuspended in 12 mM sodium phosphate buffer (pH 7.3, 650 mM NaCl 5 mM DTTand 10% glycerol). The cells were lysed by sonication on ice and centrifuged (39,000 g), and the supernatant was loaded onto tandem GSTrap (5 mL; GE Healthcare) columns. The column was washed with 12 mM sodium phosphate buffer (pH 7.3, 1.15 M NaCl and 5 mM DTT), and the protein was eluted with 12 mM sodium phosphate buffer (pH 7.3, 650 mM NaCl, 5 mM DTT and 50 mM reduced glutathione). The protein was finally purified by gel-filtration (HiLoad 26/60 superdex 200) column chromatography with 12 mM sodium phosphate buffer (pH 7.3, 650 mM NaCl, and 5 mM DTT).

### NMR experiments

We used STD and WaterLOGSY bulk ligand observation experiments. Rapid exchange of the magnetization-transferred bound ligand in a protein with the bulk of unbound ligand was observed via saturated protein signals in STD and water signals in WaterLOGSY. An excess amount of ligand versus the PAH1 domain was used to enable detection of the bulk of the unbound ligand by 1D-NMR. Because the PAH1 domain of mSin3B alone was too small to effectively transfer spin diffusions to each spin, the GST fusion system was used to increase the weight of PAH1 and effectively transfer spin diffusions to the ligand^70^.

NMR experiments were carried out at 298 K on a Bruker AVANCE700 with a cryoprobe. Spectra were processed with TOPSPIN 3.2 software. The samples for ^1^H STD and WaterLOGSY contained 10 μM GST-mSin3B PAH1 and 400 μM ligand in a volume of 500 μl in100 mM phosphate buffer (pH 7.2) containing 5% DMSO. ^1^H STD and WaterLOGSY spectra were recorded over 256 and 128 scans, respectively, in a spectral window of 8389.26 Hz centered at 3253.60 Hz. For the STD experiments, the on-resonance and off-resonance frequency of the selective pulse was switched between 30 and −0.4 ppm, respectively, after every scan.

The samples for ^1^H-^15^N HSQC titration experiments contained 100 μM mSin3B PAH1 and 100 μM (1:1 ratio) or 1 mM (1:10 ratio) ligand in 100 mM phosphate buffer (pH 7.2) containing 5% DMSO. The experiments were carried out at 298 K on a Bruker AVANCE700 with a cryo-probe. Spectra were processed with TOPSPIN 3.2 software, NMRPipe^71^ and Olivia (M. Yokochi, S. Sekiguchi, & F. Inagaki, Hokkaido University, Sapporo, Japan). In 2D ^1^H-^15^N HSQC spectra, CSPs were averaged as {(Δ*δ*^1^H)^2^ + (Δ*δ*^15^N/5)^2^}^1/2^ ppm, where Δ*δ*^1^H is the ^1^H chemical shift change and Δ*δ*^15^N is the ^15^N chemical shift change for each amino acid.

### Multivariate analysis of CSPs

For multivariate analysis, compounds with non-measurable IC_50_ above 30 μM in the DAOY cell growth inhibition experiment were classified as “ineffective”, and those with a measurable IC_50_ of 30 μM or less were classified as “effective”. The 52 compounds from *in-silico* screening with 55 variables were divided into these two groups and used as data sets. PCA and sPCA were performed by using the MixOmics^72,73^ R package; sPLS-DA and 7-fold cross validation were performed by using MetaboanalystR^74,75^ and MixOmics, respectively. In sPCA and sPLS-DA, sparse loading vectors were calculated to remove irrelevant variables by using lasso penalizations, and 10 variables were selected in each dimension. In the 7-fold cross validation, a random sort of the data set was performed 20 times and cross validation was performed for each data set by using maximum distance, centroid distance, and Mahalanobis distance prediction methods.

### NMR-guided docking calculations

NMR-guided docking calculations of the mSin3B–sertraline, mSin3B–chlorpromazine and mSin3B–YN3 complexes were performed by using HADDOCK/CNS protocols^49–51^. For the initial structures, we used a complex structure of the mSin3B PAH1 domain (PDB code: 2CZY) and energy-minimized structures of sertraline, chlorpromazine and YN3, which were generated and energy-minimized by using Avogadro^76^ with the UFF^77^ force field. For the sertraline docking calculation, Phe48, Met62, Phe65, Lys66, Ile70, Thr72, Val75, and Phe96 of mSin3B were defined as active residues, and neighboring residues were automatically defined as passive residues. Similarly, for chlorpromazine, Thr39, Leu59, Glu60, Met62, Lys66, Val91, and Phe96 of mSin3B were defined as active, and neighboring residues were defined as passive; and for YN3, Leu38, Ile46, Met62, Arg78, Phe83, Phe96, Leu97, and Tyr101 of mSin3B were defined as active, and neighboring residues were defined as passive. The rigid-body docking was performed with 1000 structures, the best 200 of which were refined first via semi-flexible molecular dynamics and then in explicit water. Root-mean-square-deviation (RMSD)-base clustering was performed with a cutoff of 2 Å. The NMR-guided docking structures were finally grouped in 11 clusters for sertraline, chlorpromazine and YN3, which comprised 72.5%, 50% and 63% of the water refinement structures, respectively. The top cluster of sertraline docking was cluster 1 with a HADDOCK score of –39.6 ± 1.0, cluster size of 48, RMSD of 1.4 ± 0.1 Å, and Z-score of −1.7. The top cluster of chlorpromazine was cluster 3 with a HADDOCK score of −41.5 ± 1.5, cluster size of 18, RMSD of 0.4 ± 0.3 Å, and Z-score of −1.8. The top cluster of YN3 was also cluster 1 with a HADDOCK score of −36.2 ± 1.9, cluster size of 31, RMSD of 1.5 ± 0.0 Å, and Z-score of −2.3.

### Cell culture and cancer sphere assay

The DAOY medulloblastoma cell line was obtained from the American Type Culture Collection and cultured in Dulbecco’s Modified Eagle’s medium (DMEM) high glucose with L-glutamine, supplemented with 10% fetal bovine serum (GIBCO Lot1706567), 50 U/ml of penicillin, and 50 μg/ml of streptomycin (Sigma), unless otherwise specified. The DAOY cells were cultured in an incubator at 310 K in a 5% CO_2_/ 5% O_2_/90% N_2_ atmosphere with maximum humidity. They were seeded at a density of 1 × 1000 cells in 100 μl in the above medium in ultralow attachment 96-well U bottom plates (SUMILON, Prime Surface MS-9096U). Assessment of spheroid size and morphology was monitored over time in a non-destructive way by using a Cell3iMager scanner (SCREEN Holdings, Kyoto, Japan). Sphere growth was scored as volume sum (volume and optical density of the single sphere).

## Electronic supplementary material

Supplementary Information

## Data Availability

The datasets generated during the current study are available from the corresponding author on reasonable request.
