# Supplementary material for: Sertraline, chlorprothixene, and chlorpromazine characteristically interact with the REST-binding site of the corepressor mSin3, showing medulloblastoma cell growth inhibitory activities
Source: Sci Rep. 2018 Sep 13;8:13763. doi: 10.1038/s41598-018-31852-1 (PMC6137095; doi:10.1038/s41598-018-31852-1)
Supplement: Supplementary file 1 — Supplementary Information [file 41598_2018_31852_MOESM1_ESM.pdf]

Supplementary Information

Sertraline, chlorprothixene, and chlorpromazine characteristically interact with the REST-binding site of the corepressor mSin3, showing medulloblastoma cell growth inhibitory activities.

Jun-ichi Kurita[1], Yuuka Hirao[1], Hirofumi Nakano[2], Yoshifumi Fukunishi[3,4], and Yoshifumi Nishimura[1]*

[1] Graduate School of Medical Life Science, Yokohama City University, 1-7-29 Suehiro-cho, Tsurumi-ku, Yokohama 230-0045, Japan
[2] Laboratory for Chemistry and Life Science, Institute of Innovative Research, Tokyo Institute of Technology, 4259 Nagatsuda-cho, Midori-ku, Yokohama 226-8503, Japan
[3] Molecular Profiling Research Center for Drug Discovery (molprof), National Institute of Advanced Industrial Science and Technology (AIST), 2-3-26, Aomi, Koto-ku, Tokyo 135-0064, Japan
[4] Technology Research Association for Next-Generation Natural Products Chemistry, 2-3-26, Aomi, Koto-ku, Tokyo 135-0064, Japan

*Corresponding author: nisimura@tsurumi.yokohama-cu.ac.jp

Supplementary Figures S1-S7 and Supplementary Table S1.

1	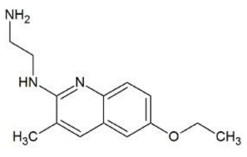	2	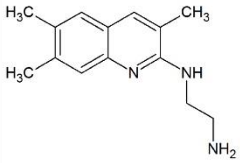	3	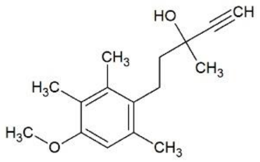
4	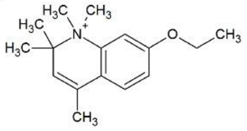	5	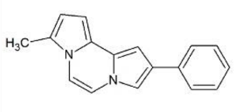	6	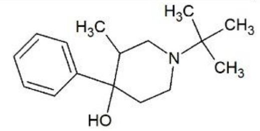
7	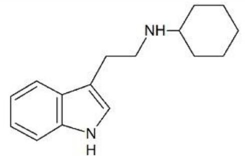	8	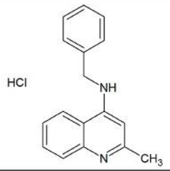	9	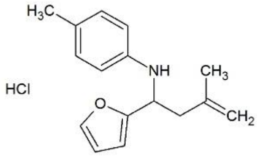
10	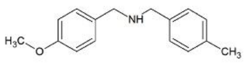	11	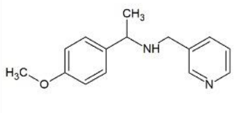	12	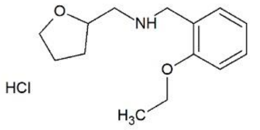
13	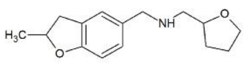	14	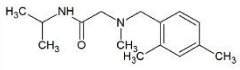	15	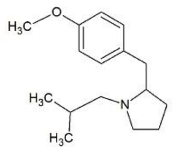
16	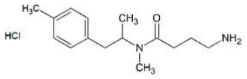	17	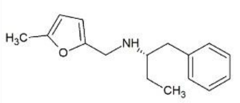	18	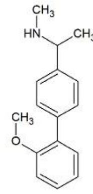
19	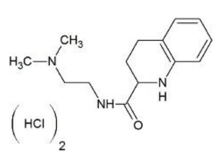	20	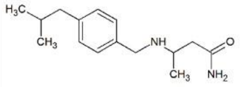	21	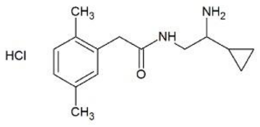
22	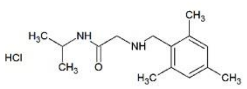	23	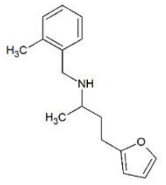	24	Eslicarbazepine acetate 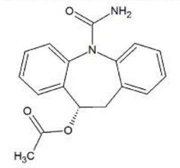

25	Mirtazapine<br>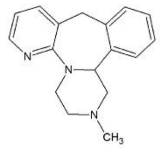	26	Apomorphine<br>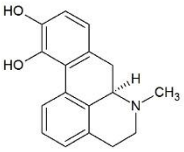	27	Bupivacaine<br>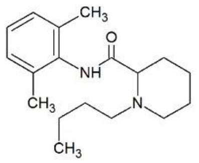
28	Chlorprothixene<br>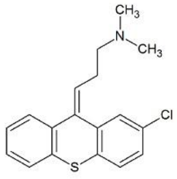	29	Chlorpromazine<br>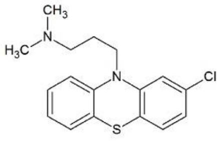	30	Biperiden<br>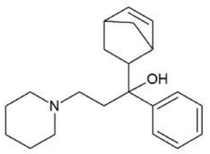
31	Sertraline<br>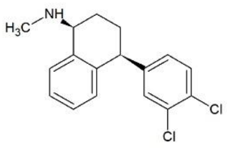	32	Rasagiline<br>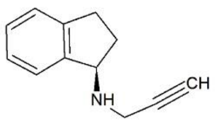	33	Oxcarbazepine<br>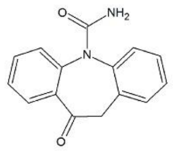
34	Galantamine<br>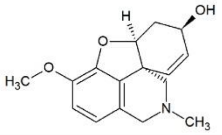	35	Ezogabine<br>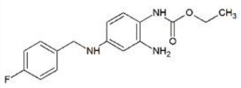	36	Rivastigmine<br>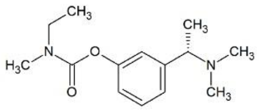
37	Carbamazepine<br>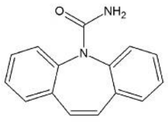	38	Tetrabenazine<br>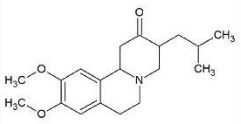	39	Capsaicin<br>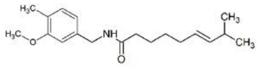
40	Maprotiline<br>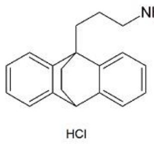	41	Naloxone<br>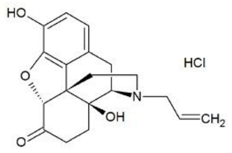	42	Amoxapine<br>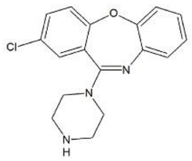

43	Zonisamide	44	Gabapentin Enacarbil	45	Fluvoxamine
	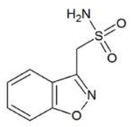		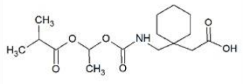		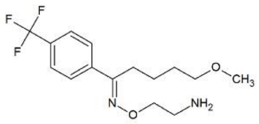
46	Carbidopa	47	Zolmitriptan	48	Melatonin
	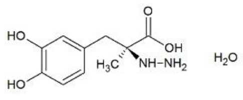		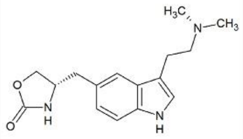		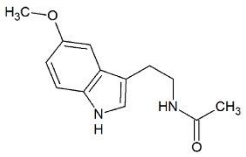
49	Glycopyrrolate	50	Scopolamine	51	Venlafaxine
	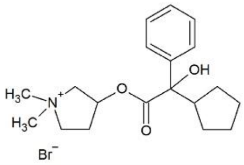		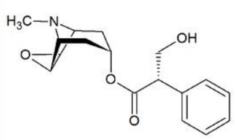		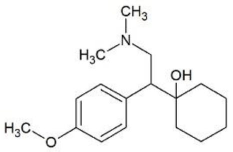
52	Atropine				
	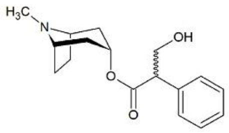				

Supplementary Figure S1. Chemical structures of 52 screened compounds

Each number “n” for each compound in this figure (1-52) corresponds to the compound name “YNn” in the main text.

a) Amide signal assignments of mSin3B PAH1 domain

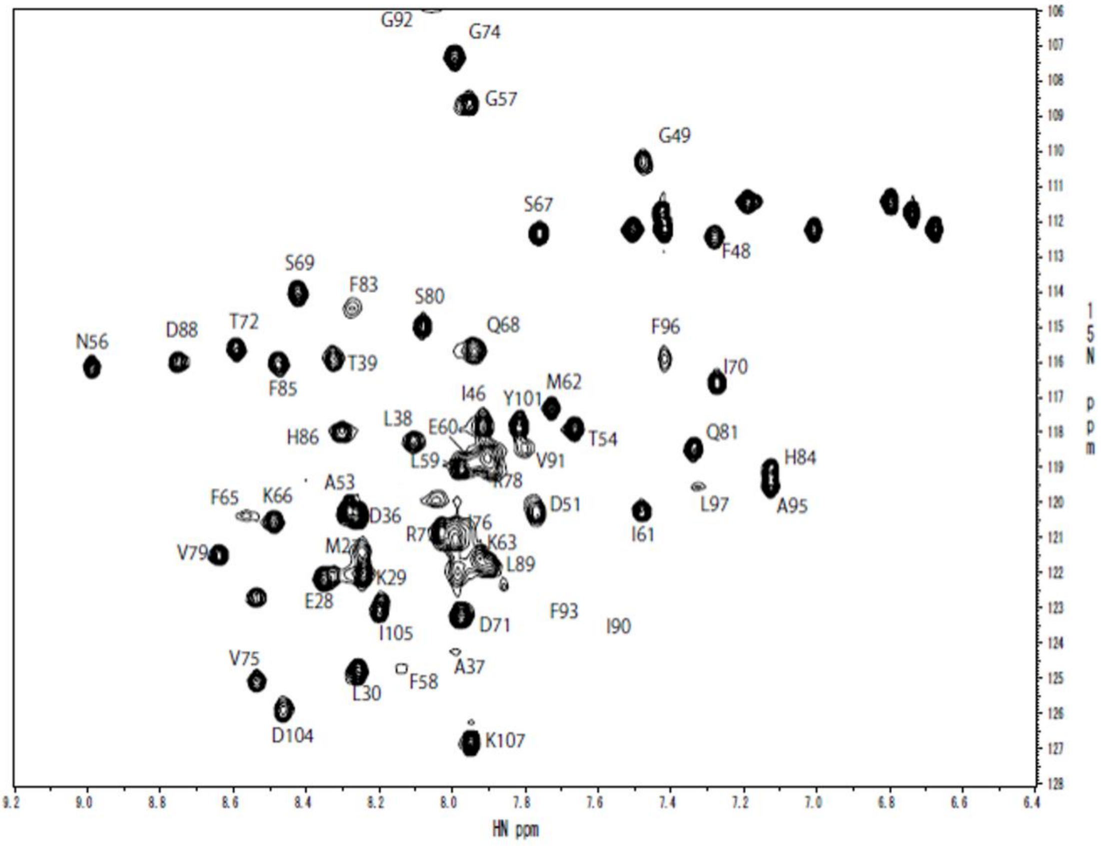

b) HSQC ligand titration experiments

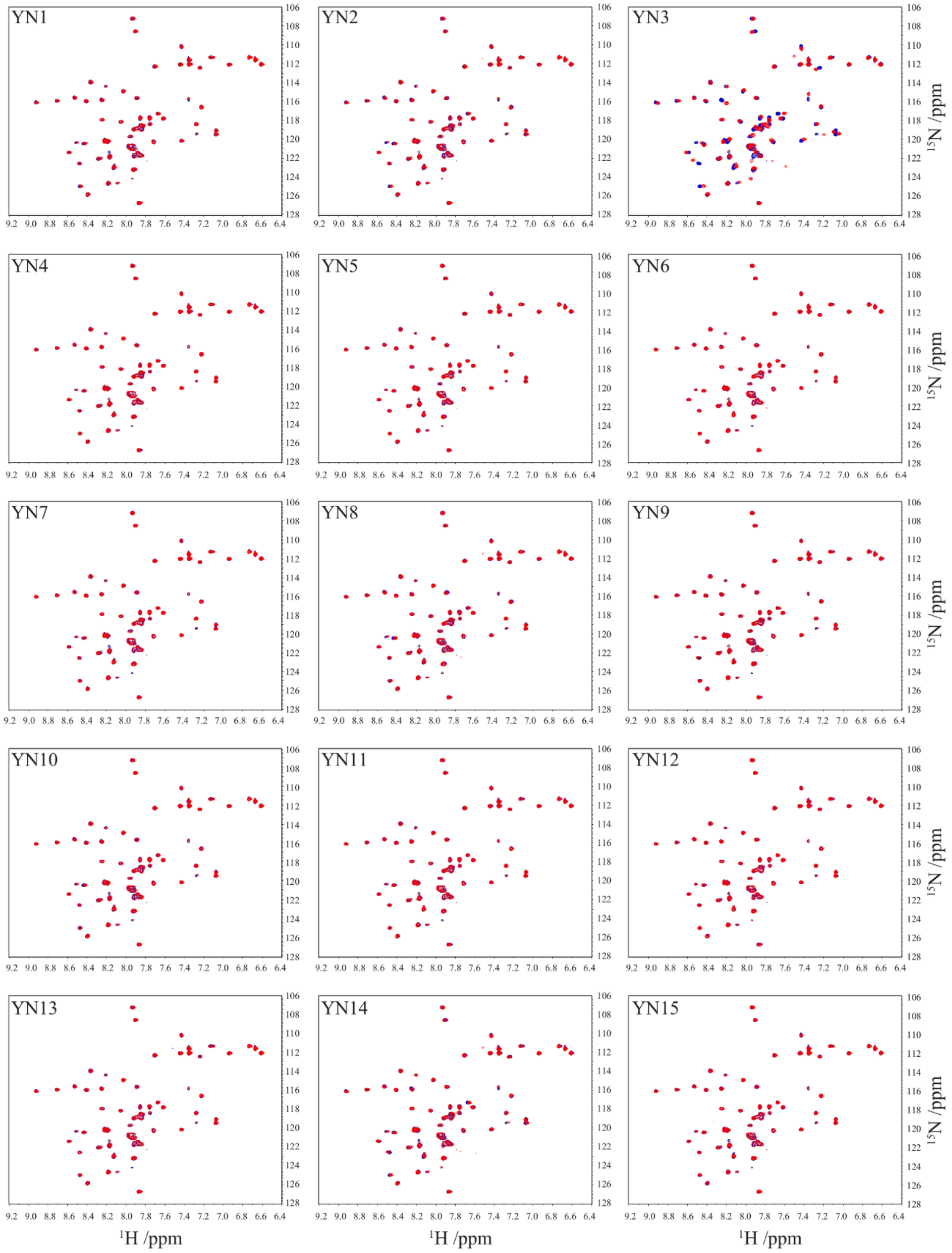

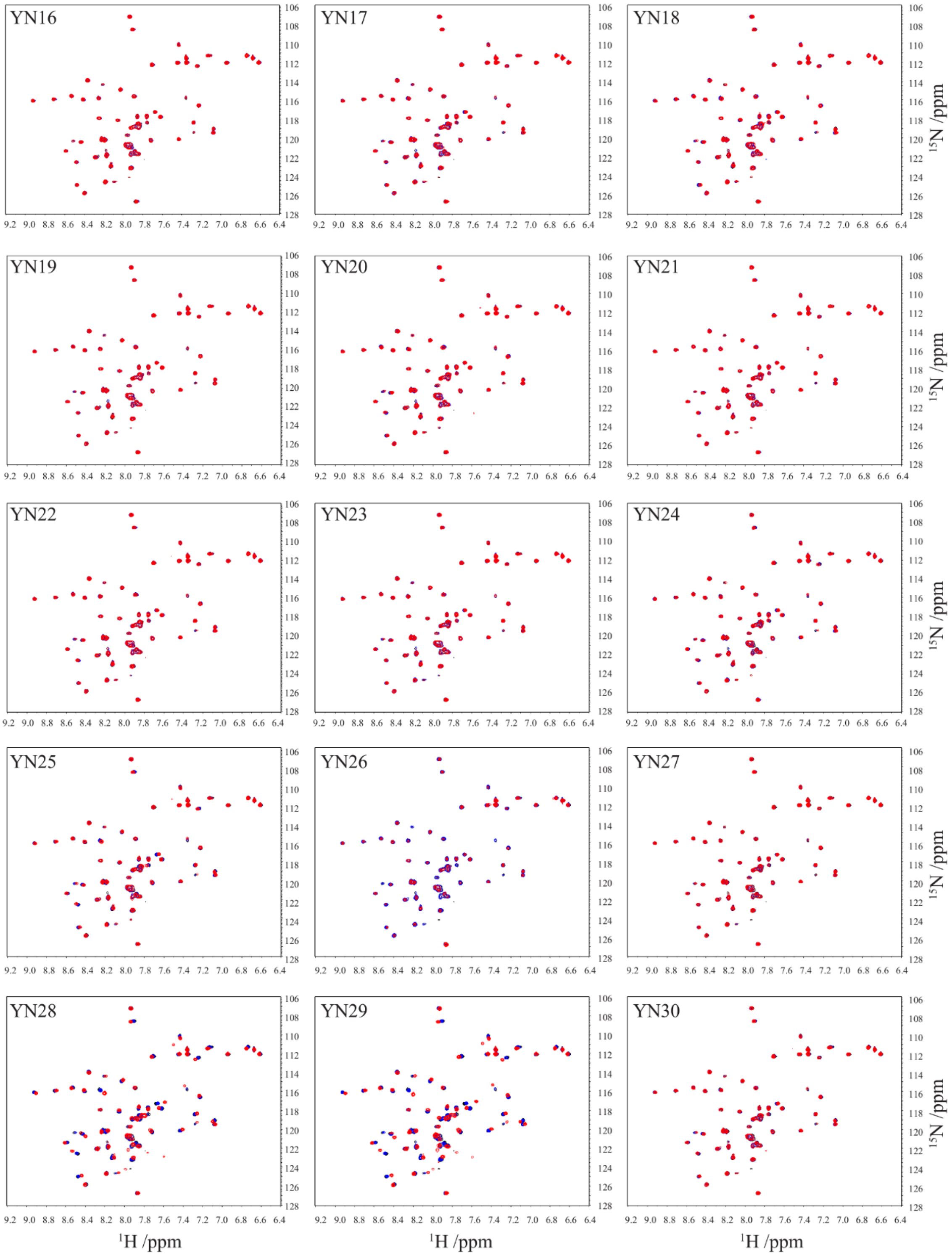

YN16
YN17
YN18
YN19
YN20
YN21
YN22
YN23
YN24
YN25
YN26
YN27
YN28
YN29
YN30
9.2 9.0 8.8 8.6 8.4 8.2 8.0 7.8 7.6 7.4 7.2 7.0 6.8 6.6 6.4
106 108 110 112 114 116 118 120 122 124 126 128
^{1}H /ppm
^{15}N /ppm

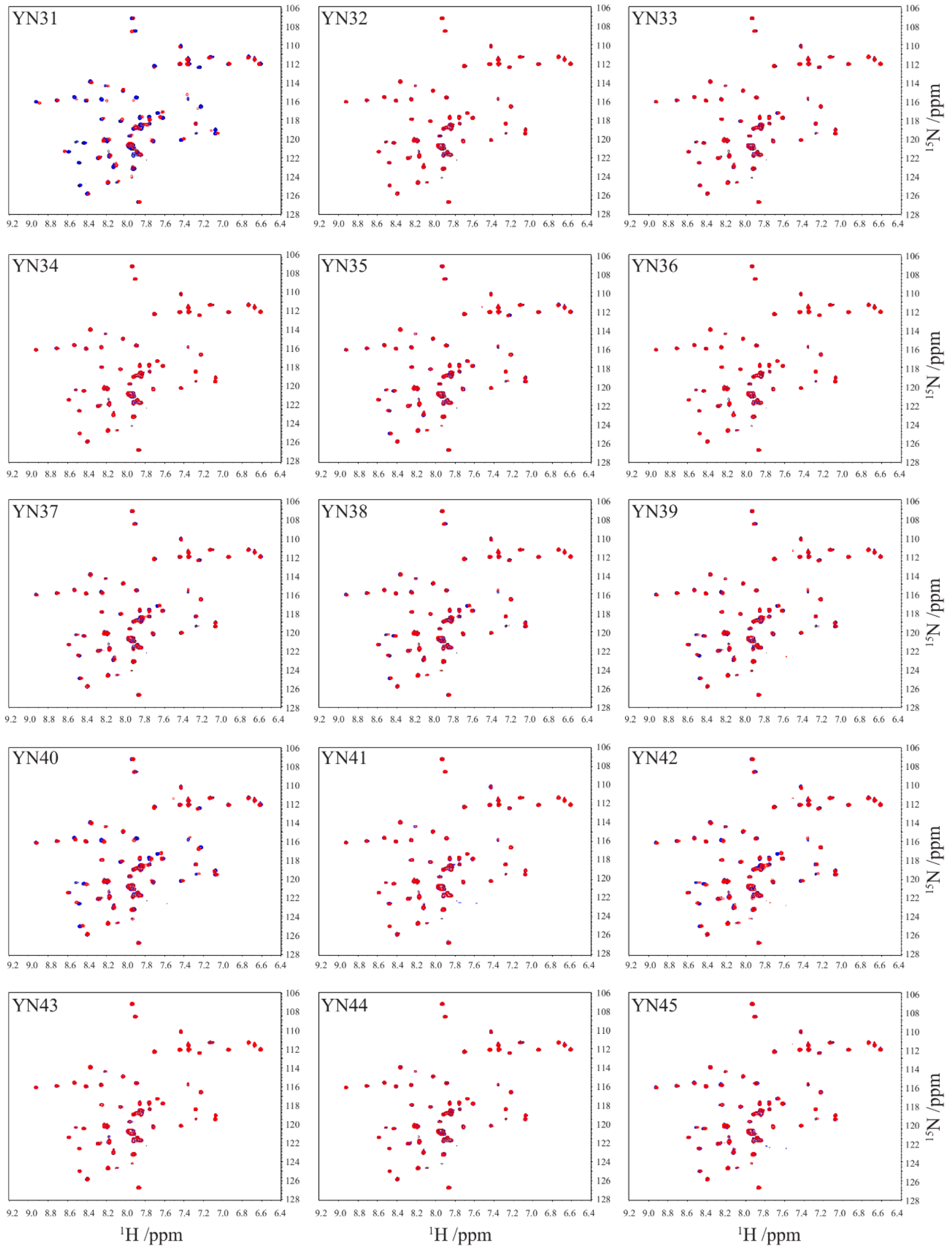
YN31
YN32
YN33
YN34
YN35
YN36
YN37
YN38
YN39
YN40
YN41
YN42
YN43
YN44
YN45
^{1}H /ppm
^{15}N /ppm

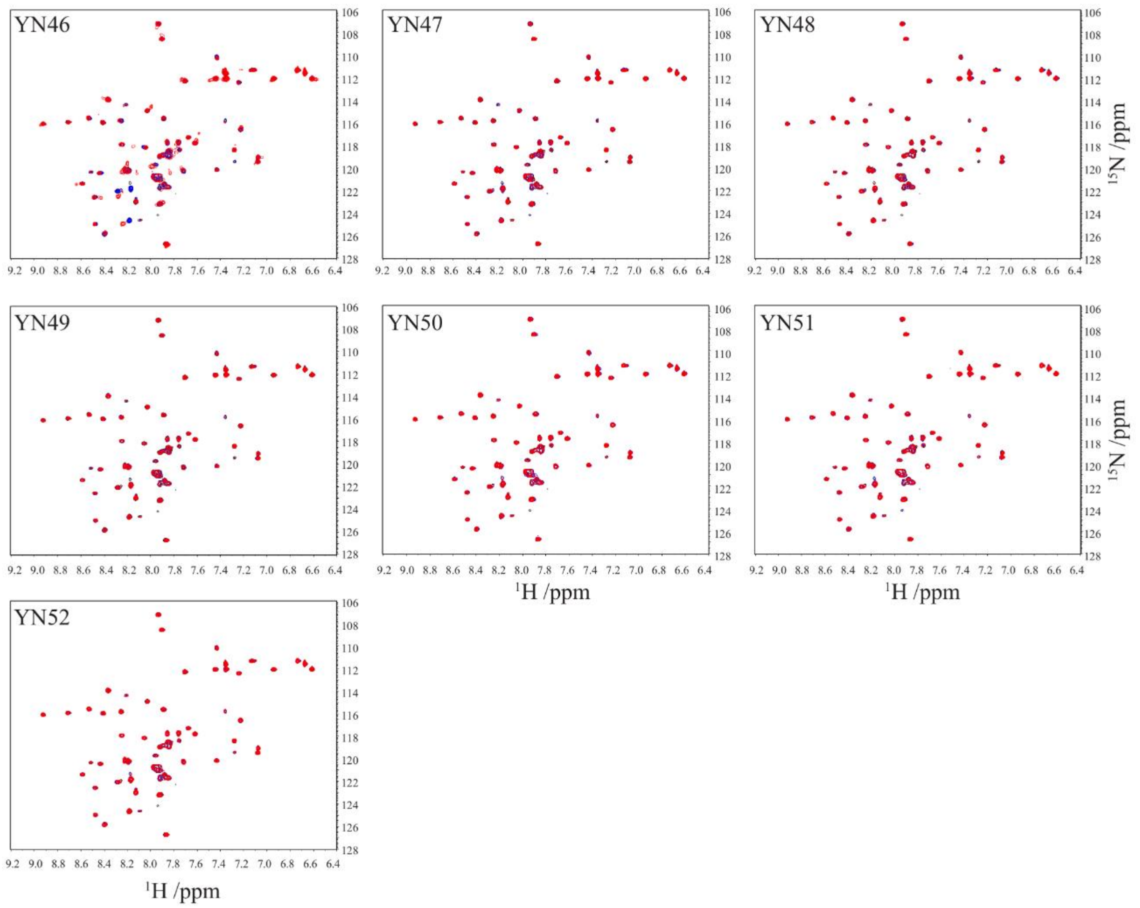

YN46
YN47
YN48
YN49
YN50
YN51
YN52
^{1}H /ppm
^{15}N /ppm

c) waterLOGSY

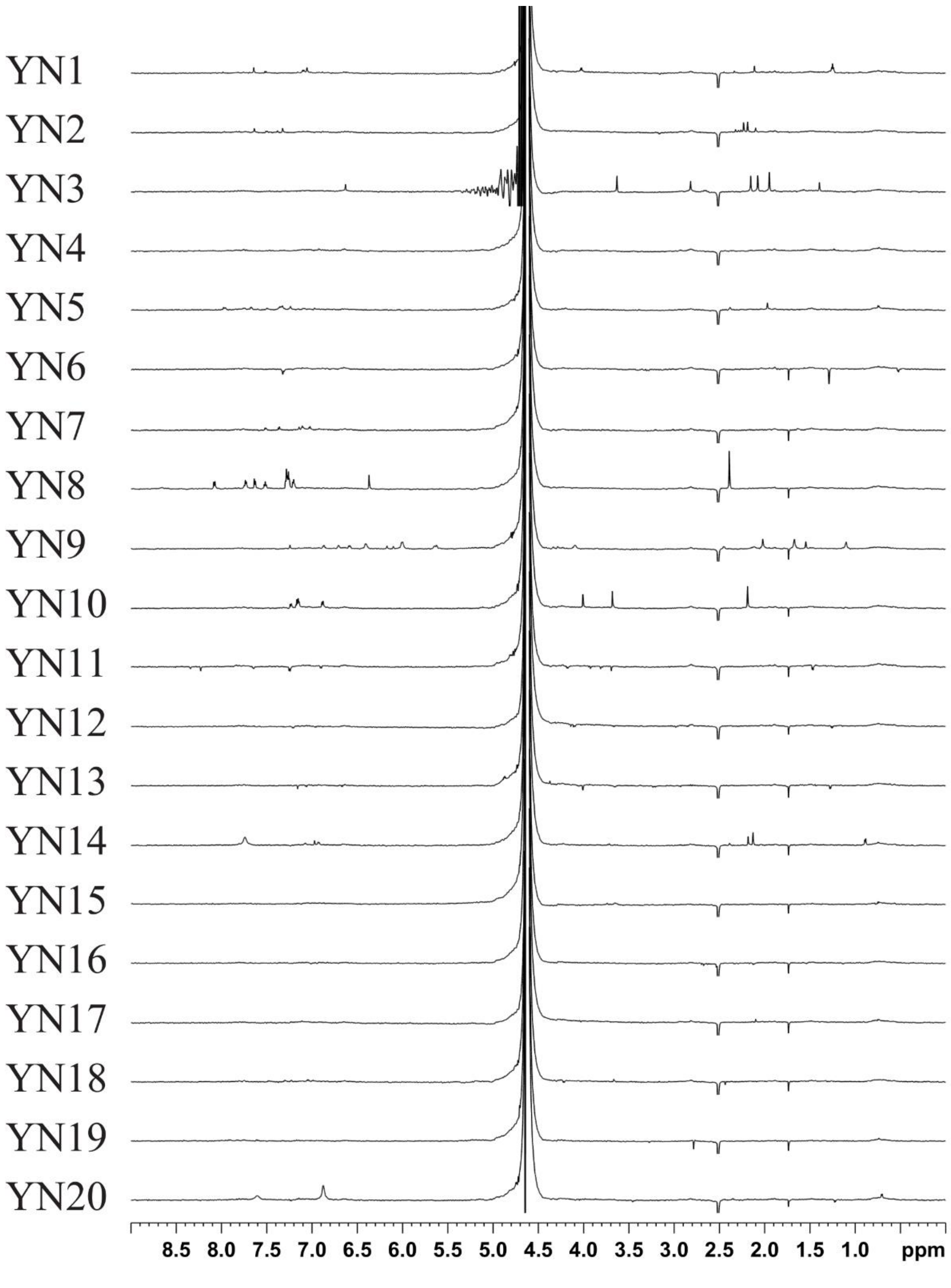

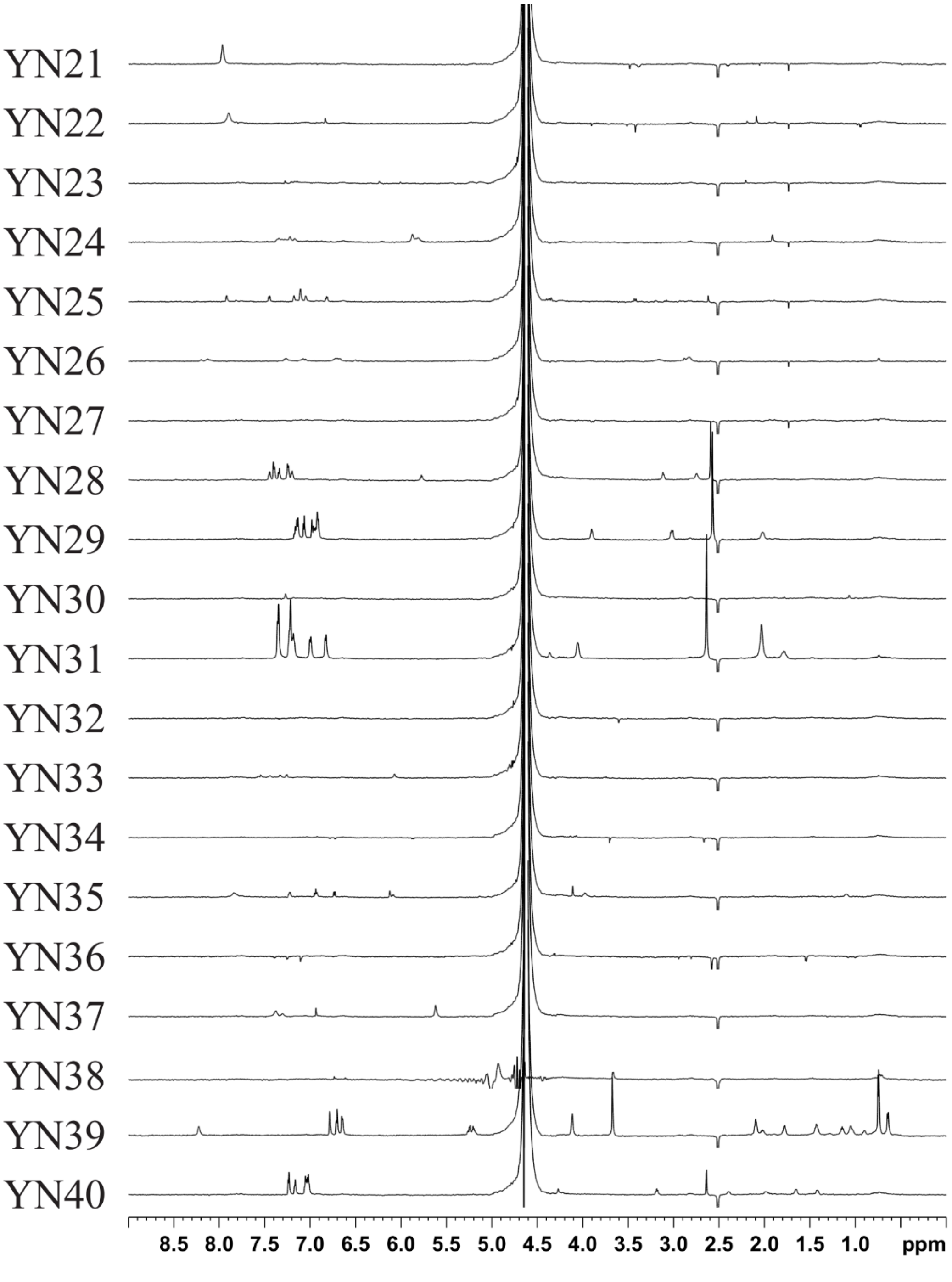
YN21
YN22
YN23
YN24
YN25
YN26
YN27
YN28
YN29
YN30
YN31
YN32
YN33
YN34
YN35
YN36
YN37
YN38
YN39
YN40
8.5 8.0 7.5 7.0 6.5 6.0 5.5 5.0 4.5 4.0 3.5 3.0 2.5 2.0 1.5 1.0 ppm

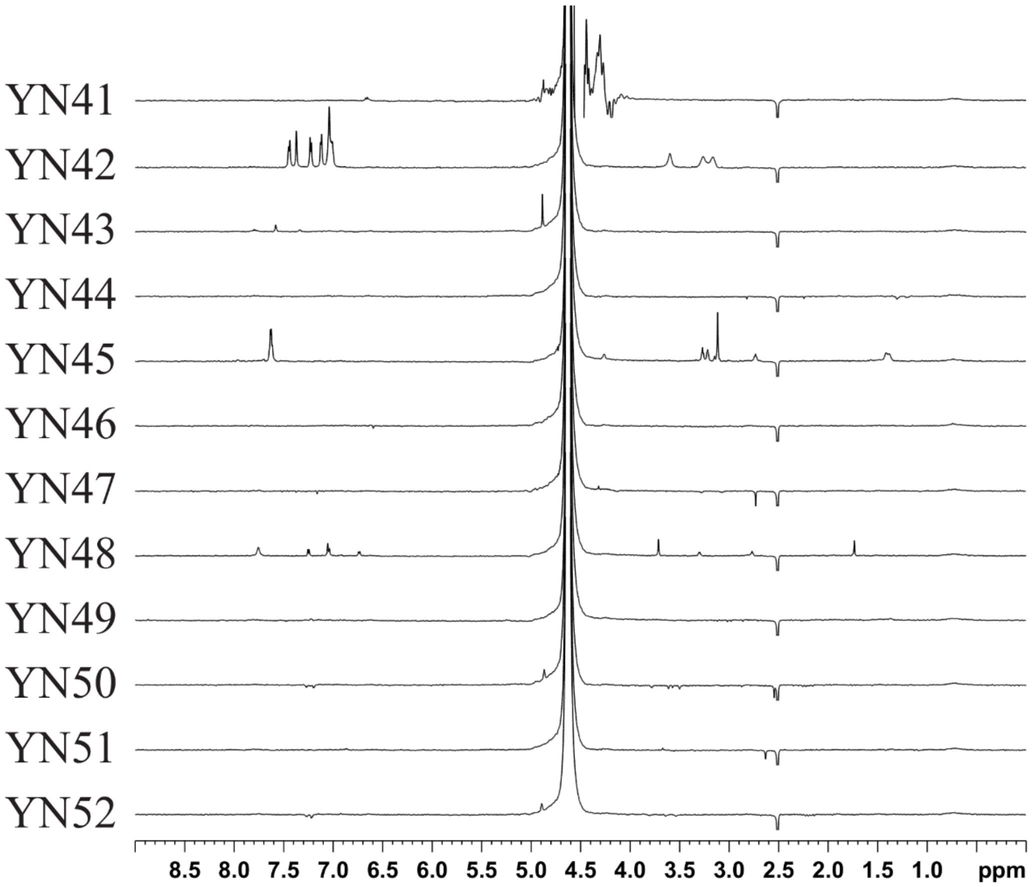
YN41
YN42
YN43
YN44
YN45
YN46
YN47
YN48
YN49
YN50
YN51
YN52
8.5 8.0 7.5 7.0 6.5 6.0 5.5 5.0 4.5 4.0 3.5 3.0 2.5 2.0 1.5 1.0 ppm

d) STD

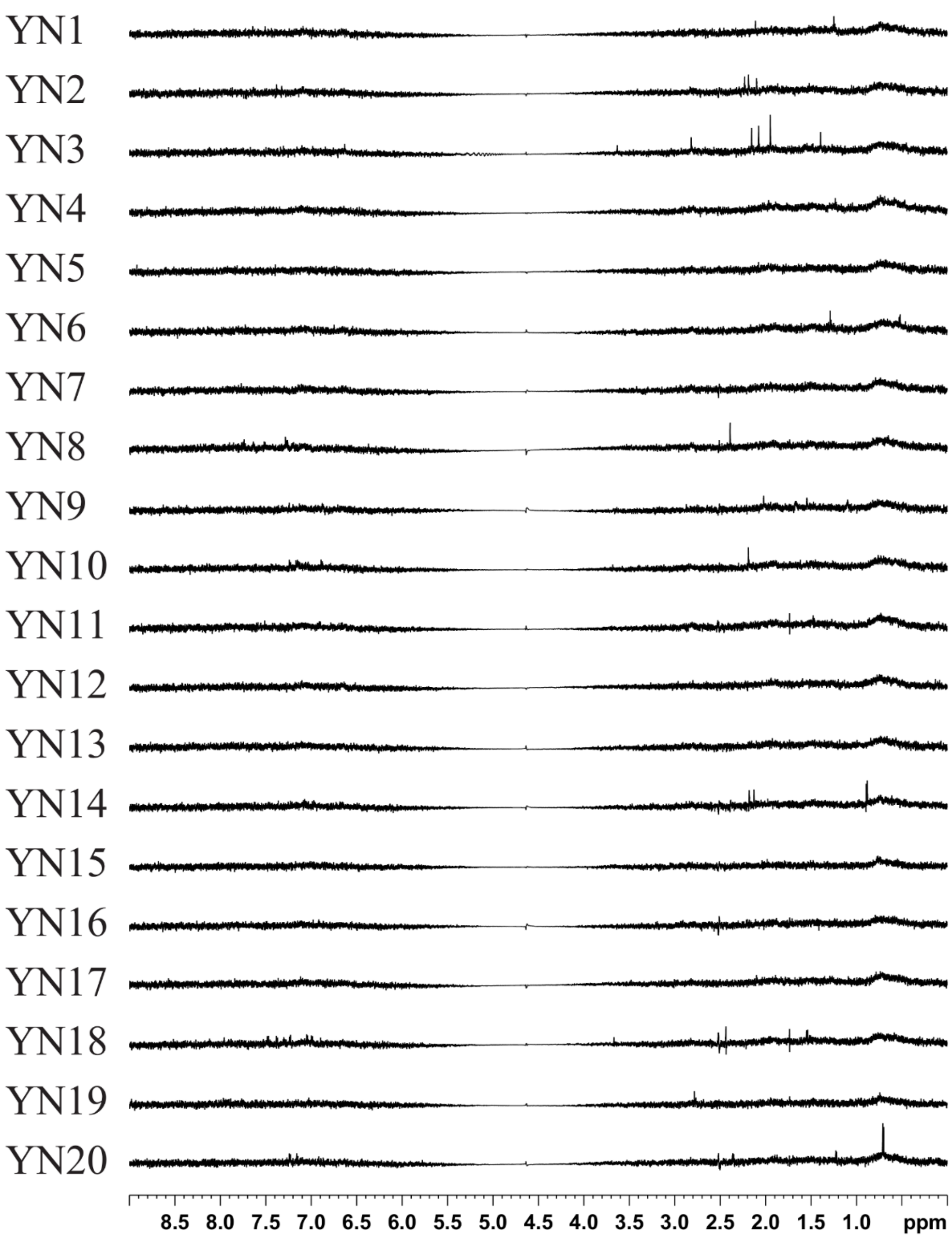
YN1
YN2
YN3
YN4
YN5
YN6
YN7
YN8
YN9
YN10
YN11
YN12
YN13
YN14
YN15
YN16
YN17
YN18
YN19
YN20
8.5 8.0 7.5 7.0 6.5 6.0 5.5 5.0 4.5 4.0 3.5 3.0 2.5 2.0 1.5 1.0 ppm

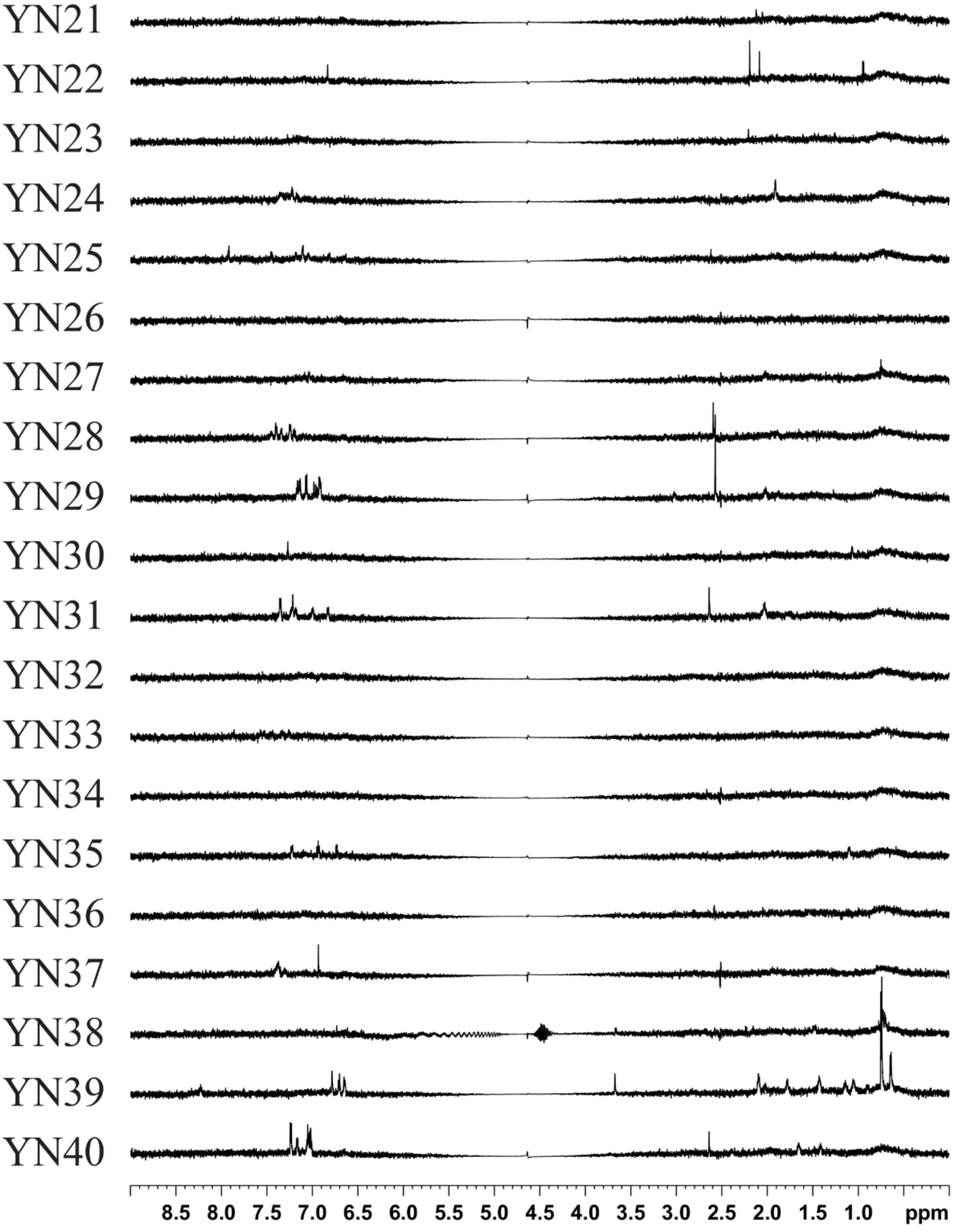
YN21
YN22
YN23
YN24
YN25
YN26
YN27
YN28
YN29
YN30
YN31
YN32
YN33
YN34
YN35
YN36
YN37
YN38
YN39
YN40
8.5 8.0 7.5 7.0 6.5 6.0 5.5 5.0 4.5 4.0 3.5 3.0 2.5 2.0 1.5 1.0 ppm

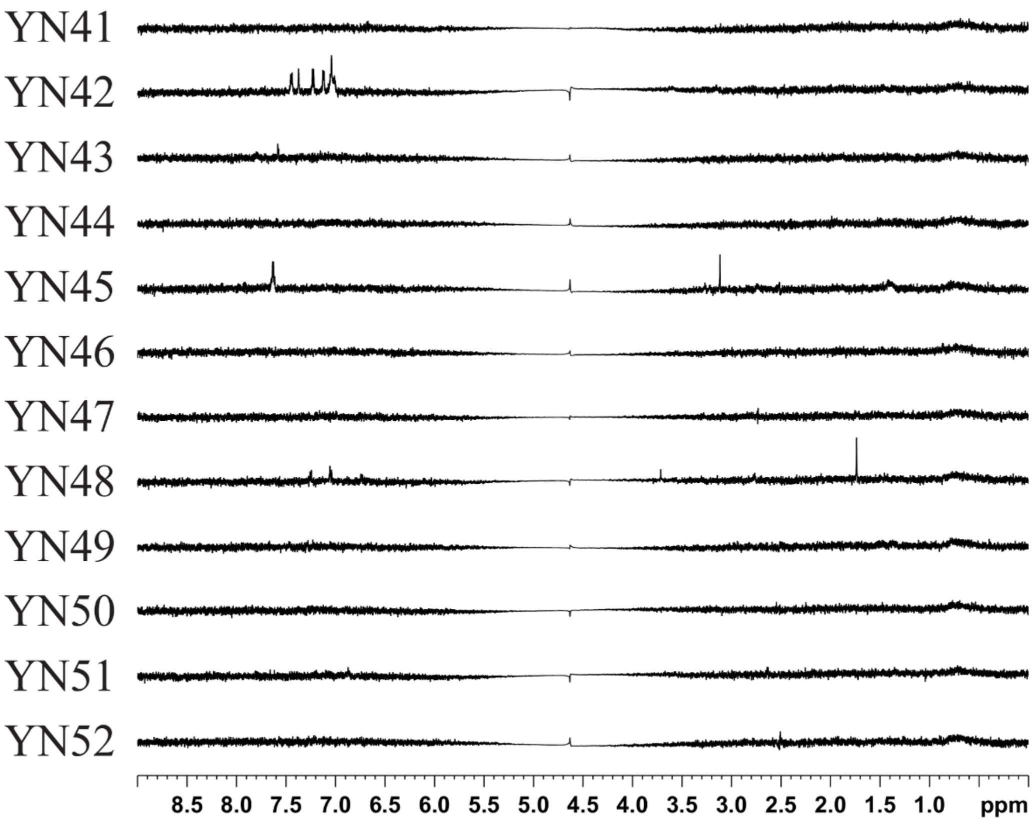
YN41
YN42
YN43
YN44
YN45
YN46
YN47
YN48
YN49
YN50
YN51
YN52
8.5 8.0 7.5 7.0 6.5 6.0 5.5 5.0 4.5 4.0 3.5 3.0 2.5 2.0 1.5 1.0 ppm

e) WaterLOGSY titration experiments

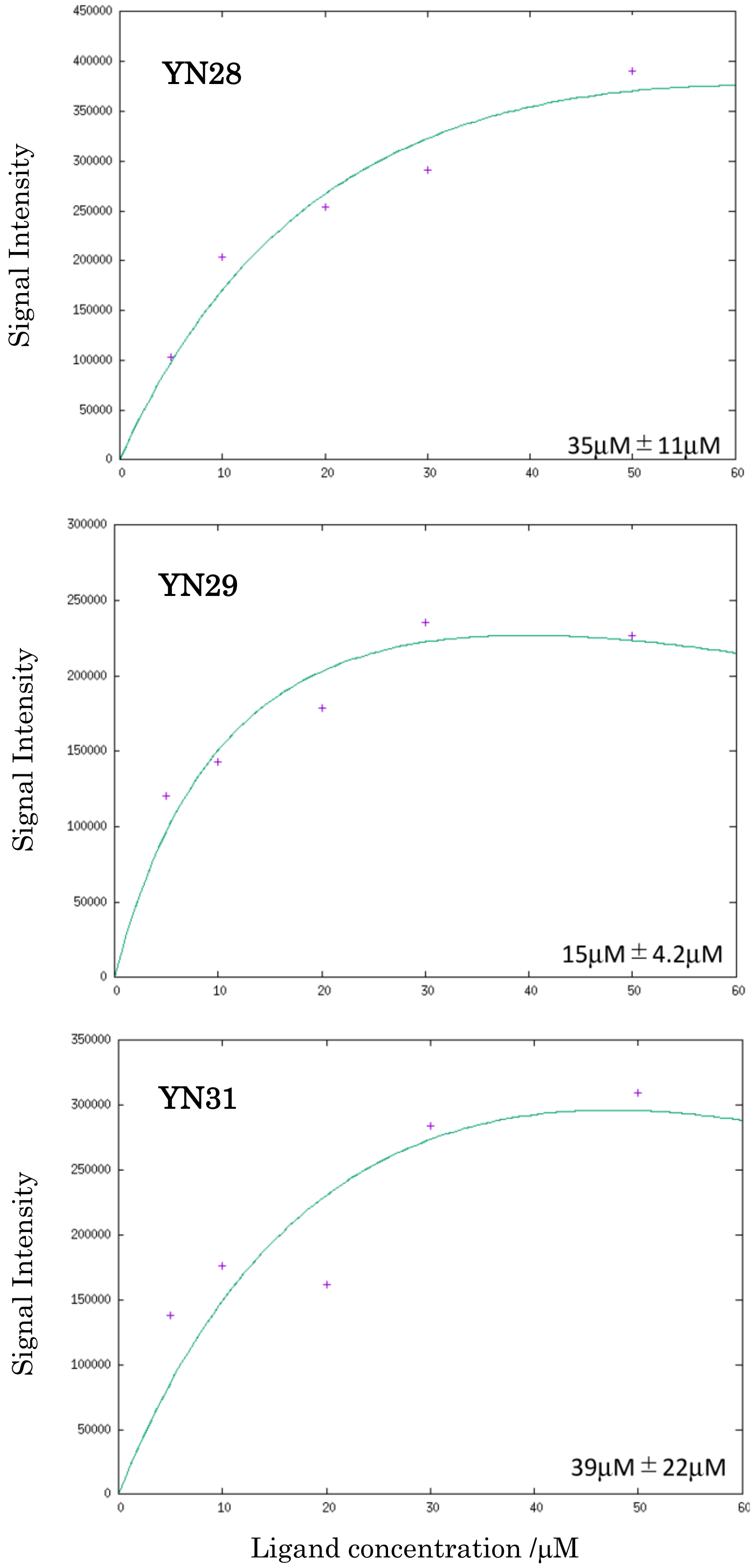

Supplementary Figure S2. HSQC ligand titration experiments, waterLOGSY and STD spectrum of each compound. a) ^{1}H-^{15}N HSQC spectrum of mSin3B PAH1 unbound form with amino acid assignments. b) HSQC ligand titration experiment of mSin3B PAH1 with each compound. In each HSQC spectrum, black signals correspond to 100 μM free PAH1 domain, and blue and red signals correspond to the additions of 100 μM and 1 mM each ligand, respectively. c) WaterLOGSY spectrum of each compound. d) STD spectrum of each compound. e) WaterLOSY titration curves. Curves were fitted for YN28, YN29, and YN31. The y axis shows signal intensity of the methyl group of each ligand in each WaterLOGSY spectrum measured and analyzed under same conditions; the x axis shows ligand concentration. To fit each titration curve of intensity depending on ligand concentration, we used the following equation: Intensity = I_{max}-$\{I_{max}/(1+L/Kd)\}$-aL, where I_{max} is the maximum WaterLOGSY signal, Kd is the dissociation constant and L is the free ligand concentration; the negative component in the equation was estimated from a WaterLOGSY spectrum measured separately at a ligand concentration of 100 μM. The equation was taken from the following reference: Dalvit, C., Fogliatto, G., Stewart, A., Veronesi, M. & Stockman, B. WaterLOGSY as a method for primary NMR screening: practical aspects and range of applicability. *J. Biomol. NMR* **21,** 349–359 (2001).

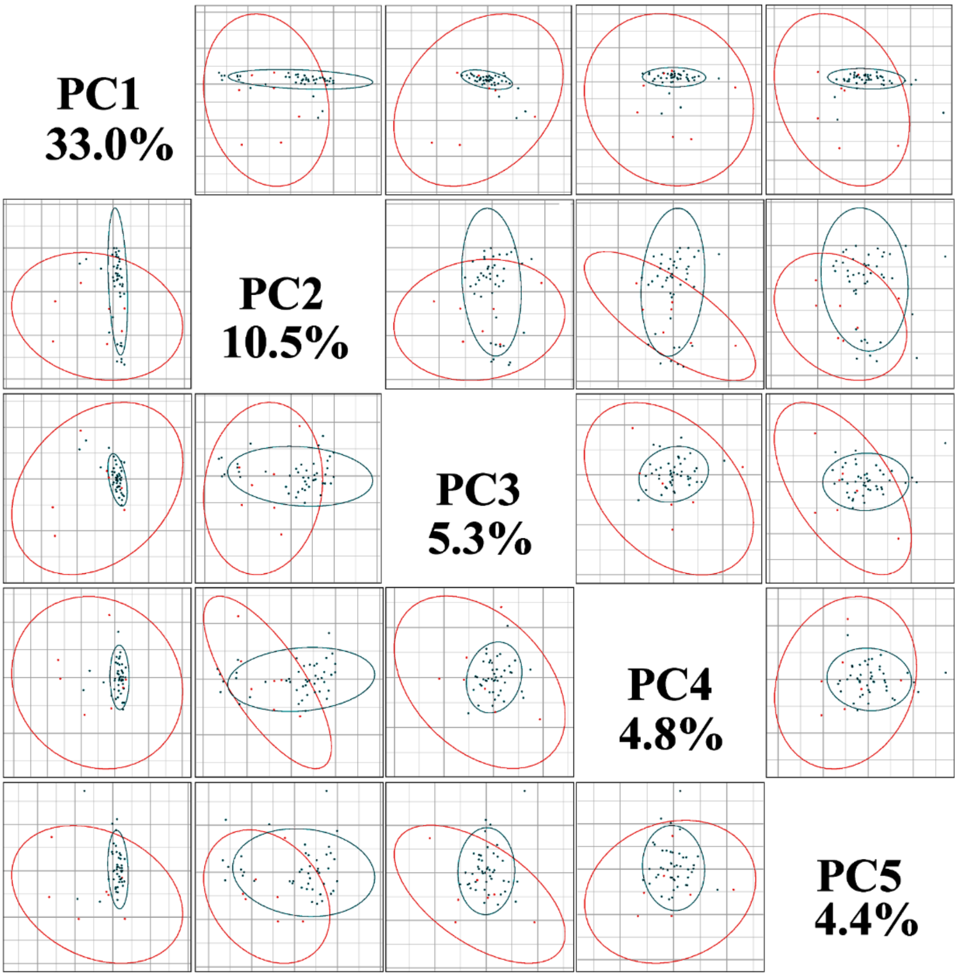

Supplementary Figure S3. Overview of pairwise score plots from the first five components

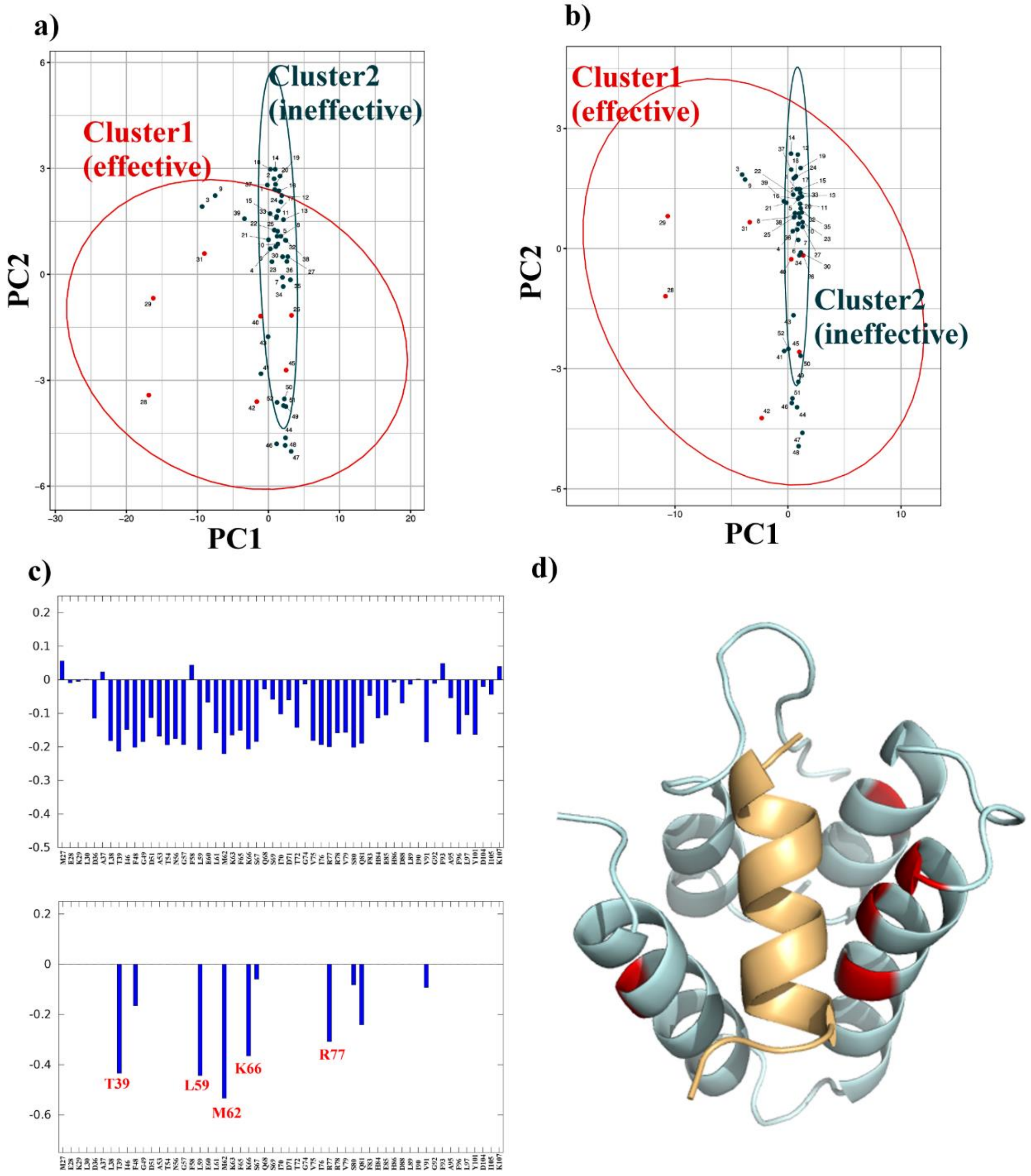

Supplementary Figure S4. Multivariate analysis of CSPs in unsupervised way and structural mapping of its loadings. a) 2D score plot of PCA applying to CSPs. Red and green solid circles indicate, respectively, the ineffective and effective compounds for DAOY cell growth inhibition. Red and green open ellipses indicate 95% confidence ellipses for the ineffective and effective compounds, respectively. b) 2D score plot of sPCA applying to CSPs. Red and green solid circles indicate, respectively, the ineffective and effective compounds for DAOY cell growth inhibition. Red (cluster1) and green (cluster2) open ellipses indicate 95% confidence ellipses for the ineffective and effective compounds, respectively. c) Loadings of PCA (upper) and sPCA (lower). The first five

sparse loadings of sPCA are represented with amino acid residues colored in red. d) Structural mapping of sparse loadings on mSin3B PAH1–REST/NRSF complex (PDB code: 2CZY). Residues corresponding to the first five sparse loadings are colored in red and the helix of REST/NRSF is colored in thin-orange.

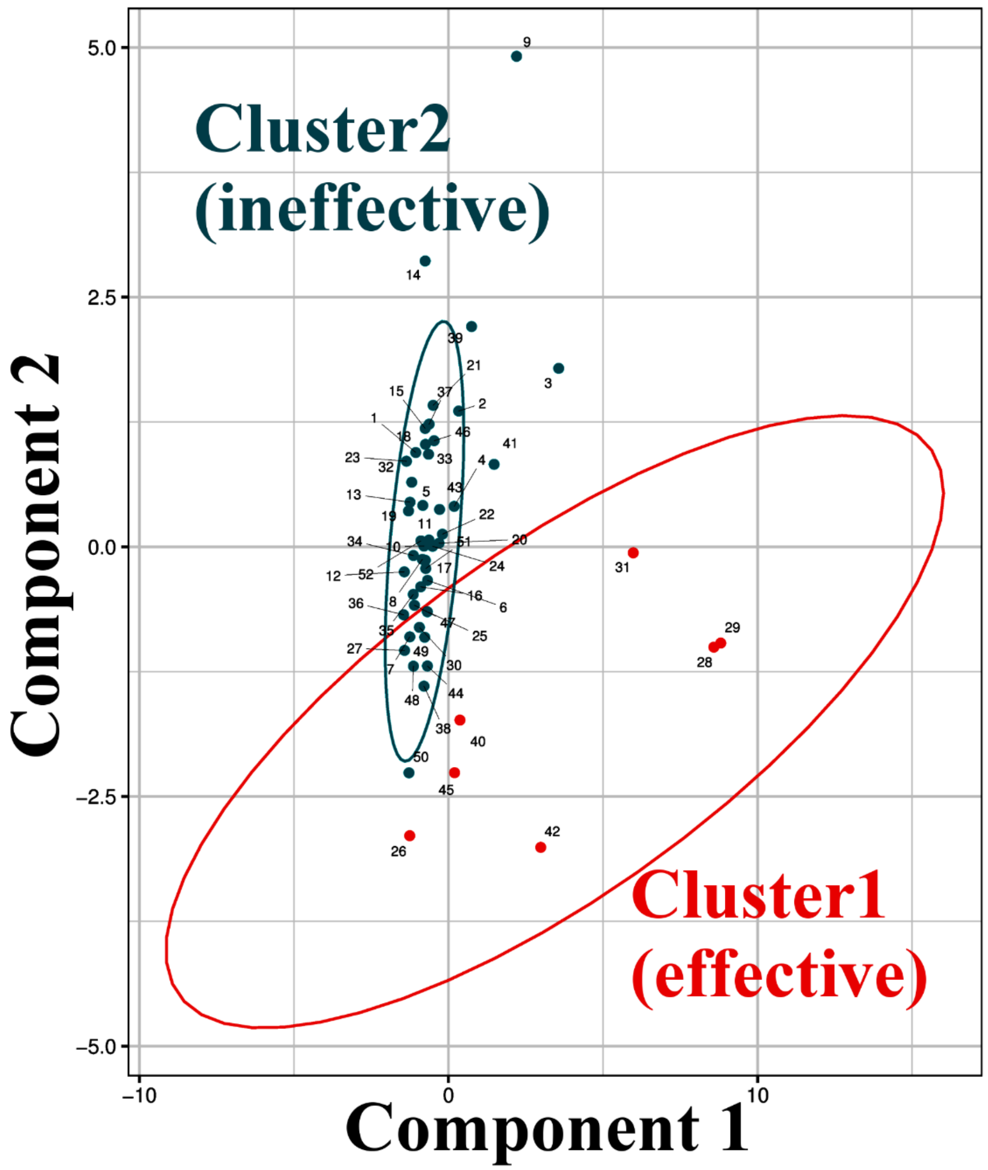

Supplementary Figure S5.

2D score plot of sPLS-DA. Red and green solid circles indicate, respectively, the ineffective and effective compounds for DAOY cell growth inhibition. Red and green open ellipses indicate 95% confidence ellipses for the ineffective and effective compounds, respectively.

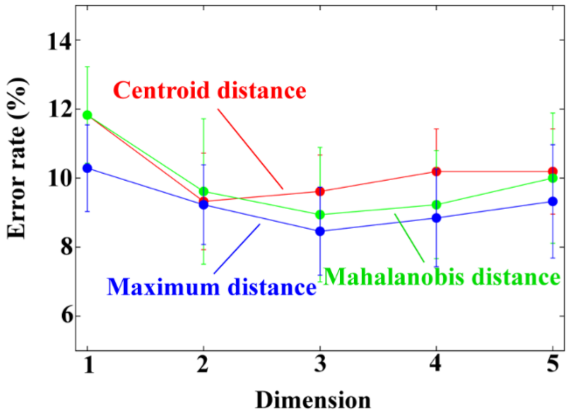

Supplementary Figure S6. 7-fold cross validation with three different distance prediction methods. Blue line, red line, and orange line indicate, respectively, maximum distance, centroids distance, and Mahalanobis distance from 7-fold cross validations of average of 20 trials

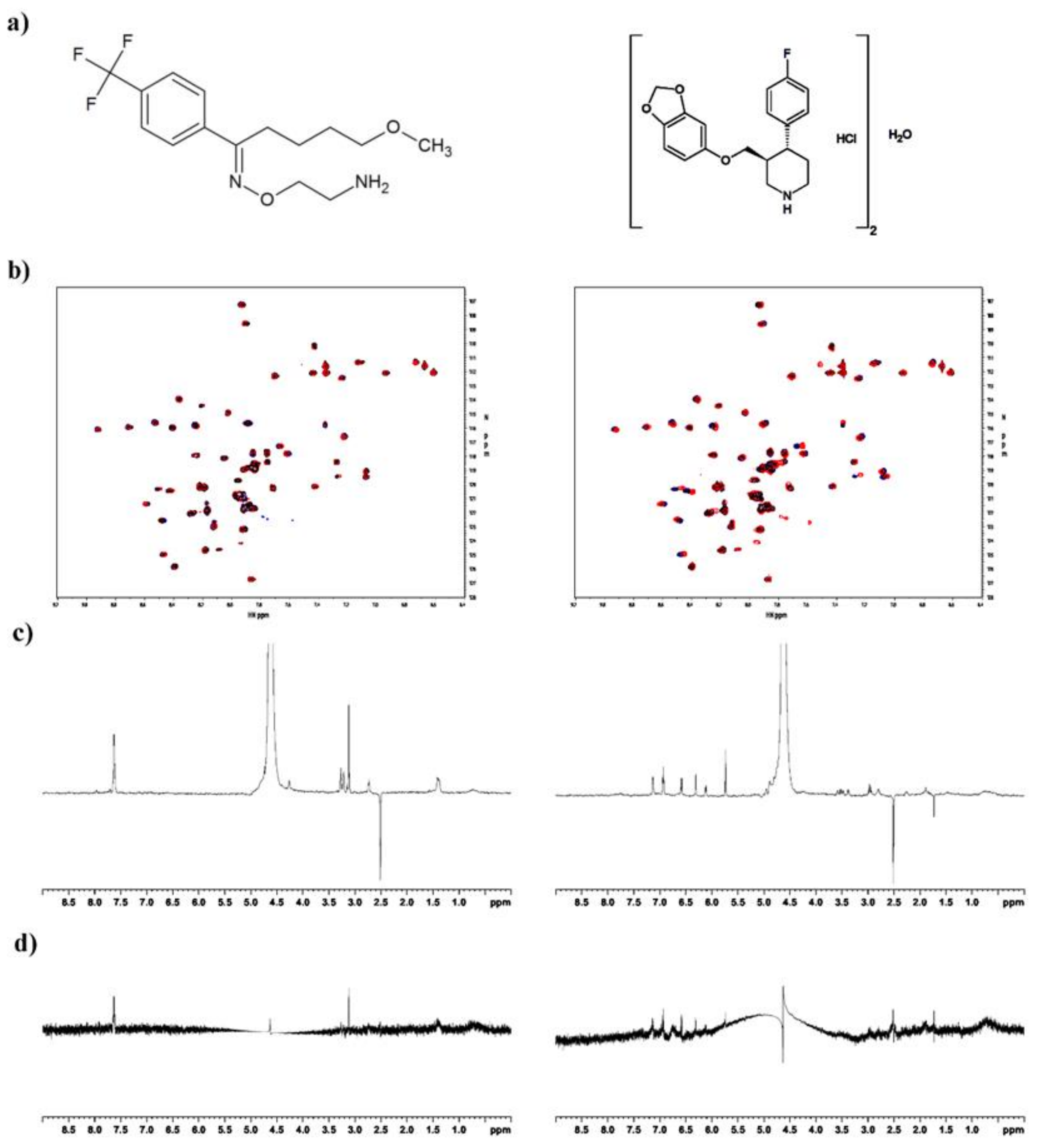

Supplementary Figure S7. NMR spectrum of fluvoxamine and paroxetine

a) Chemical structures of fluvoxamine (left) and paroxetine (right). b) HSQC ligand titration experiments of fluvoxamine (left) and paroxetine (right). In each HSQC spectrum, black signals correspond to 100 µM free PAH1 domain, and blue and red signals correspond to the additions of 100 µM and 1 mM each ligand, respectively. c) WaterLOGSY spectra of fluvoxamine (left) and paroxetine (right). d) STD spectra of fluvoxamine (left) and paroxetine (right).

Supplementary Table S1. List of compounds.

No	Supplyer	Product No	Generic name	Molecular fomula	Molecular weight (Da)	CAS	Salt	Mass Weight (Da)	Purity
1	Asinex	ASN 17325344		C14H19N3O	245.3			245.3	> 90%
2	Asinex	ASN 17325346		C14H19N3	229.3			229.3	> 90%
3	Asinex	BAS 00293111		C16H22O2	246.3			246.3	> 90%
4	Asinex	BAS 00757600		C16H24NO	246.4		I-	373.3	> 90%
5	Asinex	BAS 00903382		C17H14N2	246.3			246.3	> 90%
6	Asinex	BAS 01103140		C16H25NO	247.4			247.4	> 90%
7	Asinex	BAS 01124955		C16H22N2	242.4			242.4	> 90%
8	Asinex	BAS 01539762		C17H16N2	248.3		HCl	284.8	> 90%
9	Asinex	BAS 03420556		C16H19NO	241.3		HCl	277.8	> 90%
10	Asinex	BAS 04881724		C16H19NO	241.3			241.3	> 90%
11	Asinex	BAS 06532005		C15H18N2O	242.3			242.3	> 90%
12	Asinex	BAS 07571677		C14H21NO2	235.3		HCl	271.8	> 90%
13	Asinex	BAS 16150141		C15H21NO2	247.3			247.3	> 90%
14	Enamine	Z90241933		C15H24N2O	248.4			248.4	> 90%
15	Enamine	Z644986708		C16H25NO	247.4			247.4	> 90%
16	Enamine	Z1450403283		C15H25ClN2O	248.3		HCl	284.8	> 90%
17	Enamine	PB1730220886		C15H19NO2	245.3			245.3	> 90%
18	Enamine	EN300-86129		C16H19NO	241.3			241.3	> 90%
19	Enamine	Z300071414		C14H23Cl2N3O	229.3		(HCl)2	302.3	> 90%
20	Enamine	Z1480751560		C15H24N2O	248.4			248.4	> 90%
21	Enamine	Z1154677160		C15H23ClN2O	246.3		HCl	282.8	> 90%
22	Enamine	Z90609862		C15H25ClN2O	248.3		HCl	284.8	> 90%
23	Enamine	Z86196496		C16H21NO	243.4			243.4	> 90%
24	CHEMEXPRESS	HY-B0703	eslicarbazepine acetate	C17H16N2O3	296.3	236395-14-5		296.3	> 95%
25	CHEMEXPRESS	HY-B0352	Mirtazapine	C17H19N3	265.4	85650-52-8		265.4	> 95%

26	CHEMEXPRESS	HY-12723A	Apomorphine	C17H17NO2	267.3	58-00-4	HCl 0.5H2O	303.78	> 95%
27	BIONET	BS-5224	Bupivacaine	C18H28N2O	288.4	2180-92-9		288.44	>95%
28	CHEMEXPRESS	HY-B0274	Chlorprothixene	C18H18ClNS	315.9	113-59-7		315.9	> 95%
29	BIONET	KS-5101	Chlorpromazine	C17H19ClN2S	318.9	50-53-3		318.87	>97%
30	AKSCI	P695	Biperiden	C21H29NO	311.5	514-65-8		311.5	98%
31	ENAMINE	EN300-150167	Sertraline	C17H17Cl2N	306.2	79617-96-2	HCl	342.691	95%
32	BIONET	FS-3130	rasagiline mesylate	C12H13N	171.2	136236-51-6		171.24	>95%
33	TCI	O0363	Oxcarbazepine	C15H12N2O2	252.3	28721-07-5		252.27	>98.0%
34	CHEMEXPRESS	HY-76299	Galantamine	C17H21NO3	287.4	357-70-0		287.4	> 95%
35	BIONET	KE-0201	Ezogabine	C16H18FN3O2	303.3	150812-12-7		303.34	>97%
36	CHEMEXPRESS	HY-17368	Rivastigmine	C14H22N2O2	250.3	123441-03-2		250.3	> 95%
37	CHEMEXPRESS	HY-B0246	Carbamazepine	C15H12N2O	236.3	298-46-4		236.3	> 95%
38	TCI	T2839	Tetrabenazine	C19H27NO3	317.4	58-46-8		317.43	>99.0%
39	CHEMEXPRESS	HY-10448	Capsaicin	C18H27NO3	305.4	404-86-4		305.4	> 95%
40	ENAMINE	EN300-226729	Maprotiline	C20H23N	277.4	10262-69-8	HCl	313.864	95%
41	CHEMEXPRESS	HY-17417	Naloxone	C19H21NO4	327.4	465-65-6	HCl	363.8	> 95%
42	Sigma	A129	Amoxapine	C17H16ClN3O	313.8	14028-44-5		313.33	> 95%
43	TCI	Z0026	Zonisamide	C8H8N2O3S	212.2	68291-97-4		212.22	>98.0%
44	CHEMEXPRESS	HY-A0057A	Gabapentin Enacarbil	C16H27NO6	329.4		HCl	207.7	> 95%
45	AKSCI	O893	Fluvoxamine	C15H21F3N2O2	318.3	54739-18-3		318.3	98%
46	KEMPROTEC	CAS 28860-95-9	Carbidopa	C10H14N2O4.H2O	244.2	38821-49-7		244.24	> 95%

47	TCI	Z0024	Zolmitriptan	C16H21N3O2	287.4	139264-17-8		287.36	>98.0%
48	CHEMEXPRESS	HY-B0075	Melatonin	C13H16N2O2	232.3	73-31-4		232.3	> 95%
49	TCI	G0392	Glycopyrrolate	C19H28NO3+	318.4	596-51-0	Br-	398.34	>98.0%
50	CHEMEXPRESS	HY-N0296A	Scopolamine	C17H21NO4	303.4	51-34-3		384.3	> 95%
51	TORONTO	V119995	Venlafaxine	C17H27NO2	277.4	93413-69-5		277.4	> 95%
52	ALFA AESAR	J64677	Atropine	C17H23NO3	289.4	51-55-8		289.37	>99%
